# CYSLTR1 antagonist inhibits Th17 cell differentiation by regulating the NF-κB signaling for the treatment of psoriasis

**DOI:** 10.7150/ijbs.92514

**Published:** 2024-03-25

**Authors:** Junpeng Zhao, Yi Chen, Liming Li, Huiqi Yin, Shasha Song, Yongfang Wang, Xiwei Feng, Xinyu Fan, Changxing Gao, Lingyu Gao, Yijing Zhan, Ming Zhao, Xinyu Li, Qianjin Lu

**Affiliations:** 1 Hospital for Skin Diseases, Institute of Dermatology, Chinese Academy of Medical Sciences and Peking Union Medical College, Nanjing, China.; 2 Peking Union Medical College, Chinese Academy of Medical Sciences, Beijing, China.; 3 Key Laboratory of Basic and Translational Research on Immune-Mediated Skin Diseases, Chinese Academy of Medical Sciences, Nanjing, China.; 4 Jiangsu Key Laboratory of Molecular Biology for Skin Diseases and STIs, Institute of Dermatology, Institute of Dermatology, Chinese Academy of Medical Sciences and Peking Union Medical College, Nanjing, China.

**Keywords:** Montelukast, CYSLTR1, Psoriasis, Th17 cell, NF-κB signaling

## Abstract

Cysteinyl leukotriene receptor 1 (CYSLTR1) is observed to increase in psoriatic skin lesions. Montelukast, a CYSLTR1 antagonist, effectively treats inflammatory disorders, such as rheumatoid arthritis, multiple sclerosis, and atopic dermatitis. Thus, blocking CYSLTR1 may be a promising strategy for psoriasis immunotherapy. We prepared a montelukast sodium cream and solution and investigated their effects on psoriasis-like skin lesions induced by imiquimod (IMQ). After the treatment, serum, skin, and spleen samples were collected for evaluation. We treated human T helper (Th) 17 cells with montelukast *in vitro* to study its effect on Th17 differentiation and nuclear factor kappa-B (NF-κB) signaling. We also created a keratinocyte proliferation model induced by M5 cytokines and assessed the influence of montelukast on key psoriasis-related genes. We induced psoriasis in CYSLTR1 knockout (KO) mice using IMQ to explore the role of CYSLTR1 in psoriasis development. Montelukast sodium cream and solution effectively reduced the psoriasis area and severity index (PASI) and alleviated disease symptoms in IMQ-induced mice. Furthermore, reduced infiltration of inflammatory cells (Th1, Th17, and T follicular helper [Tfh] cells), decreased mRNA expression of cytokines in the skin (interleukin [IL]-17/F and IL-23), and lower serum concentrations of various cytokines (IL-2, IL-6, IL-13, and IL-17A/F) were observed. Montelukast cream and solution also decreased spleen size and the proportion of Th17 and Tfh cells, and significantly inhibited NF-κB signaling-related genes after application. Moreover, montelukast inhibited Th17 cell differentiation and suppressed NF-κB signaling *in vitro*. CYSLTR1 KO mice induced with IMQ showed improvement in PASI scores, serum IL-17A/F levels, and lower Th1 and Th17 cells in the spleen and skin compared to wild-type mice. Montelukast also suppressed the proliferation and inflammatory response of keratinocytes by regulating NF-κB signaling. Collectively, our results strongly indicate that inhibition of CYSLTR1 signaling to target the Th17 response holds significant promise as a therapeutic approach to manage psoriasis.

## Introduction

Psoriasis is a chronic autoimmune disorder that manifests as enduring, non-contagious skin irregularities characterized by red, pink, or purple-colored, dry, itchy, and scaly patches 1. Severity of psoriasis ranges from localized lesions to extensive coverage. Plaque psoriasis (psoriasis vulgaris) comprises approximately 90% of cases and exhibits red patches with white scales that commonly affect the forearms, shins, navel, and scalp. Guttate psoriasis presents as drop-shaped lesions; pustular psoriasis presents as small and non-infectious pus-filled blisters; inverse psoriasis presents as red patches on the skin folds; and erythrodermic psoriasis results in a widespread rash, potentially arising from other types. Common nail psoriasis includes pits or color changes 1, 2. Psoriatic arthritis, a variable form of chronic inflammatory arthritis, frequently coexists with skin and nail psoriasis 3. The primary objectives of managing psoriasis are to alleviate symptoms, reduce inflammation, and control excessive proliferation of keratinocytes 4. Currently, various medications and therapeutic approaches are used to manage this condition. Systemic oral medications serve as the primary therapeutic approach for managing moderate-to-severe plaque psoriasis 5. Oral agents commonly used for the clinical treatment of psoriasis include acitretin, fumarates, methotrexate, cyclosporine, and apremilast. However, their clinical utility may be impeded by adverse side effects such as bone marrow suppression and potential liver and kidney toxicity 5.

In recent decades, biologics have transformed psoriasis treatment. Precision-targeted therapies, including receptor fusion proteins and recombinant monoclonal antibodies, specifically target inflammatory disorders 6. However, not all patients respond adequately and some experience reduced effectiveness over time, necessitating the development of new medications with distinct mechanisms of action. Several current systemic psoriasis treatments have potential risks, such as liver toxicity, immunosuppression, and cardiovascular side effects 7, 8. The emergence of novel therapies not only expands therapeutic choices but potentially decreases overall management costs and improves access to effective therapies for a broader population.

Cytokines from T helper (Th) 17 cells, specifically interleukin (IL)-17A, play a pivotal role in sustaining inflammation in psoriatic plaques 9. IL-17A promotes the proliferation of epidermal keratinocytes, leading to the production of several antimicrobial peptides and chemokines such as chemokine (C-X-C motif) ligand (CXCL) 1, CXCL2, CXCL8, and chemokine (C-C motif) ligand (CCL) 20. The presence of antimicrobial peptides contributes to increased skin inflammation. IL-17A upregulates chemokine and antimicrobial peptide synthesis in keratinocytes and facilitates the recruitment of inflammatory cells 10. This exacerbates skin inflammation, as CCL20, which originates from IL-17A-stimulated keratinocytes, attracts IL-17A-producing Th17 cells, perpetuating a vicious feed-forward cycle that culminates in a fully developed state of psoriasis 11.

Cysteinyl leukotriene receptor 1 (CYSLTR1) is a member of G-Protein-Coupled Receptor (GPCR) and is involved in the pathogenesis of several chronic inflammatory and immune disorders, including asthma 12, allergic rhinitis 13, rheumatoid arthritis (RA) 14, osteoarthritis 15, and multiple sclerosis (MS) 16. Montelukast is a selective antagonist of CYSLTR1, which can ameliorate leukotriene (LTD)-induced bronchoconstriction and significantly reduce both the early and late airway responses to allergens, rendering it effective against allergic rhinitis and asthma. Furthermore, montelukast has been shown to modulate Th17 migration and suppress IL-17 production, thereby delaying the onset of an experimental model mimicking MS 16. Moreover, montelukast exerts an inhibitory influence on the inflammation associated with RA by reducing the secretion of key cytokines (metalloproteinase [MMP]-3, MMP-13, IL-8, and IL-6) in fibroblast-like synoviocytes induced by IL-1β 17. Furthermore, a notably high number of CYSLTR1-positive cells has been described in the dermal tissue of patients with psoriasis than in individuals with healthy skin, underscoring the pivotal role of leukotrienes and their receptor (CYSLTR1) in psoriasis pathogenesis 18. Considering its anti-allergic and anti-inflammatory properties and significant advancements in addressing two distinct autoimmune disorders, montelukast has potential therapeutic efficacy in autoimmune diseases. However, studies on using montelukast treating psoriasis are lacking. Therefore, this study evaluated the effects and underlying mechanisms of montelukast in the treatment of psoriasis, offering a novel approach to manage autoimmune skin diseases.

## Materials and Methods

### Animal models

Eight-week-old female C57BL/6J and BALB/c mice, and B6/JGpt-Cysltr1*^em1Cd/Gpt^* (CYSLTR1 knockout [KO]) mice were obtained from GemPharmatech Co., Inc. All necessary safety measures, including autoclaving the nestlets, bedding, cages, water, and food, were undertaken to maintain sterility. Skin lesions in 9-week-old BALB/c mice and psoriasis-like models induced by external use of imiquimod (IMQ) on the skin of the BALB/c mice, which were provided by the Hospital for Skin Diseases, Institute of Dermatology, Chinese Academy of Medical Sciences. The samples were subjected to immunohistochemistry (IHC) and western blotting (WB) to assess CYSLTR1 protein expression. Our experimental procedures adhered to rigorous ethical standards for animal research and care, as previously described 19. These protocols were approved by the local government authorities to ensure strict compliance (no. 2022-DW-004).

### Human samples

Using human naïve CD4^+^ cell magnetic beads (480042, BioLegend), naïve CD4^+^ T cells were recovered from the peripheral blood mononuclear cells (PBMCs) of healthy patients. Skin samples from patients with psoriasis and healthy controls with pigmented nevi were obtained from the Pathology Department and Biobank of the Institute of Dermatology, Chinese Academy of Medical Sciences. The ethics committee of the hospital (permission ID: 2021.054) provided ethical permission for all investigations using patient samples. All participants provided written informed consent before participation. Detailed information on the skin samples is provided in Supplementary Table S1.

### Experimental design

#### BALB/c, C57BL/6J, and CYSLTR1-KO mice

**Figure 1:**
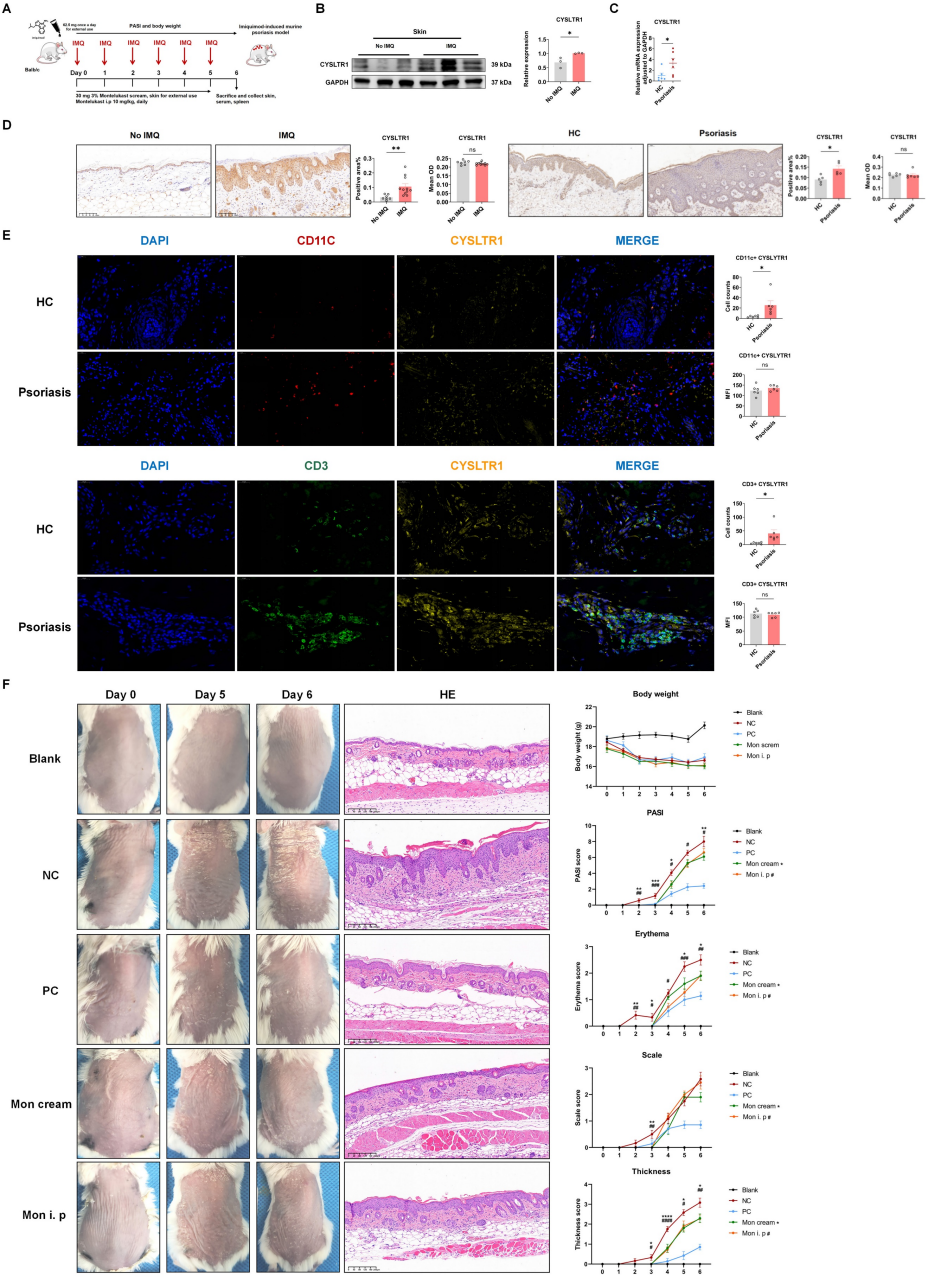
**Montelukast sodium cream ameliorates IMQ-induced psoriasis-like skin lesions in BALB/c mice. (A)** A schematic of the treatment using montelukast sodium cream and solution in IMQ-induced psoriasis-like murine model. **(B)** High expression of CYSLTR1 in IMQ-induced psoriasis-like skin lesions.** (C)** High mRNA level of *CYSLTR1* in skin lesion of patients with psoriasis. **(D)** IHC of CYSLTR1 staining in skin lesions from psoriasis murine models and psoriasis patients.** (E)** Multi-color IHC staining of CD11c^+^ CYSLTR1^+^ and CD3^+^ CYSLTR1^+^ cells in skin lesions from patients with psoriasis.** (F)** The skin lesions, histological features, and body weight, as well as the changes in PASI scores, erythema, scale, and thickness scores of the psoriasis-like mice. n = 7 in Blank group; n = 11 in IMQ + cream base (negative control: NC) group; n = 7 in IMQ + HMS (positive control: PC) group; n = 10 in IMQ + montelukast cream group; n = 11 in IMQ + montelukast intraperitoneal (i. p) injection group. Horizontal bars represent the mean ± standard error of the mean (SEM), *p < 0.05, **p < 0.01, ***p < 0.001, ****p < 0.0001.

BALB/c mice were randomized into four groups when they were 8 weeks old (blank control: n = 7, no IMQ stimulation; positive control [PC] group: n = 7, halometasone (HMS) treatment; negative control [NC]: n = 11, cream base treatment; montelukast cream group [3%]: n = 10; montelukast intraperitoneal (i. p.) injection group: n = 11). All mice were given daily topical applications of 62.5 mg 5% IMQ cream (H20030128, Sichuan Med-Shine, Chengdu, China) for six days (d) in a row on shaved dorsal skin, apart from the blank control group. The mice in the montelukast cream group received a daily dose of 30 mg, applied at 4-hour (h) interval following IMQ application, while the NC group received an equal amount of the vehicle cream base. The PC group received daily topical application of 0.05% HMS cream (Bright Future Pharmaceutical Lab. Ltd., Hong Kong, China) at 4-h intervals after IMQ application. The montelukast i. p group received a daily dose of 10 mg/kg. However, the blank control group did not undergo any external application of IMQ (Figure 1A).

To elucidate the contribution of CYSLTR1 to the pathogenesis and progression of psoriasis, we engineered knockout mice with CYSLTR1 deficiency. At 8 weeks of age, both CYSLTR1-KO (n = 5) and C57BL/6J mice (Wild-type, WT) (n = 6) were subjected to daily topical application of 5% IMQ cream continuously for 6 d, which induced psoriasis-like skin lesions (Figure 7A).

All mice were humanely sacrificed at 9 weeks of age, and their skin, serum, and spleen were collected for future studies. The spleen index was derived by dividing the organ weight by body weight. The severity of skin lesions was determined using the psoriasis area and severity index (PASI). Erythema, thickness, and scaling were evaluated from 0 (none) to 4 (very prominent). The cumulative score (range 0-12) served as an indicator of the severity of skin lesions and was computed by summing the scaling, erythema, and thickness scores 20.

### Histological evaluation

Mouse skin tissues were fixed in formalin before being embedded in paraffin (BL539A, Biosharp, Hefei, China). Subsequently, the tissue samples were sectioned into slices with a thickness of 5 µm and subjected to staining using hematoxylin and eosin. The stained sections were stored at 20-25 ℃. A digital fluorescent microscope (BX53F2; Olympus, Tokyo, Japan) was used for assessing the epidermal thickness and invading inflammatory cells.

### Immunohistochemistry

The tissue sections were incubated 12 h at 4 °C with the corresponding primary antibodies. After thoroughly washing, horseradish peroxidase-conjugated antibodies were added to the sections for 1.5 h at 24~28 ℃. Next, anti-CYSLTR1 antibodies (PH6465, Abmart, Berkeley Heights, NJ, USA) were added at a dilution of 1:200. The peroxidase activities were assessed using a TMB kit (P0211, Beyotime Biotechnology, Shanghai, China). A fluorescence microscope (Olympus BX53F2) was used to examine CYSLTR1-positive cells. The OLYMPUS cellSens Standard Imaging Software (https://www.olympus-lifescience.com/en/software/cellsens/) was used to document and analyze fluorescent staining. The mean fluorescence intensity (MFI) and positive area of CYSLTR1 staining were calculated using ImageJ version 1.8 (National Institute of Health, Bethesda, MD, USA) for quantification.

For multicolor IHC staining, the CY3-Tyramide-TSA: G1223, iF647-Tyramide-TSA: G1232, and FITC-Tyramide-TSA: G1222 kits (Wuhan Servicebio Technology Co., Ltd., Wuhan, China) were used. Briefly, sections of human skin tissues from healthy controls and patients with psoriasis were subjected to epitope retrieval in citrate buffer (G1202, Wuhan Servicebio Technology Co., Ltd.) and incubated with blocking buffer (GC305010, Wuhan Servicebio Technology Co., Ltd.,), followed by primary antibodies CYSLTR1 (PH6465, Abmart), CD3 (GB15014, Wuhan Servicebio Technology Co., Ltd.), and CD11c (GB11059, Wuhan Servicebio Technology Co., Ltd.) at 25 ℃ (30 minutes [min]). Background staining was further reduced, and the sections were rinsed with citrate buffer. Sections were scanned using a Pannoramic MIDI (3DHISTECH, Budapest, Hungary) and CaseViewer Software (https://www.3dhistech.com/solutions/caseviewer/).

The MFI of CYSLTR1 staining in CD3 and CD11c cells and the cell counts of CD3^+^ CYSLTR1^+^ and CD11c^+^ CYSLTR1^+^ cells were calculated using ImageJ version 1.8 for quantification.

### RNA-Sequencing

#### RNA isolation and library preparation

Total RNA was extracted from the skin tissues of the IMQ-induced murine psoriasis model (NC *vs* montelukast cream: 4 *vs* 4) using TRIzol reagent (Invitrogen, Carlsbad, CA, USA), following the manufacturer's guidelines. A NanoDrop 2000 spectrophotometer (Thermo Scientific, Waltham, MA, USA) and an Agilent 2100 Bioanalyzer (Agilent Technologies, Santa Clara, CA, USA) were used to assess RNA purity and quantification, respectively. Subsequent library construction was performed using the VAHTS Universal V6 RNA-seq Library Prep Kit (Vazyme biotech co., ltd., Nanjing, China) according to the manufacturer's instructions. Transcriptome sequencing and analysis were performed by OE Biotech Co., Ltd. (Shanghai, China).

#### RNA sequencing and differentially expressed genes analysis

The libraries were sequenced on an Illumina NovaSeq 6000 platform, producing 150 bp paired-end reads. The initial processing of raw reads in the FASTQ format involved fastp 21 eliminating low-quality reads to obtain a set of clean reads. Subsequent mapping of clean reads to the reference genome was performed using HISAT 22. Fragments per kilobase of transcript per million mapped reads and read counts for each gene were determined using the HTSeq-count 23. DESeq2 24. was used for differential expression analysis, with significance criteria set at Q value < 0.05, and fold change > 2 or fold change < 0.5 to identify significant differentially expressed genes (DEGs). R version 3.2.0 (R Foundation for Statistical Computing, Vienna, Austria) was used for the hierarchical cluster analysis of DEGs that showed gene expression patterns across groups and samples. A radar map of the top 30 genes was generated in R using the “Ggradar” package to depict the expression of the upregulated or downregulated DEGs.

Gene Ontology (GO) 25 and Kyoto Encyclopedia of Genes and Genomes (KEGG) 26 pathway enrichment analyses of DEGs were conducted based on a hypergeometric distribution, and R version 3.2.0 was used to create column diagrams, chord diagrams, and bubble diagrams representing significant enrichment terms.

### *In vitro* psoriatic model establishment

We established an *in vitro* model to mimic psoriasis using HaCaT keratinocytes. The cells were cultivated at 37 °C and 5% CO_2_. Dulbecco's Modified Eagle Medium (DMEM; 11960044, Gibco, Waltham, MA, USA) was used as the growth medium. To elicit an inflammatory response that closely resembles numerous aspects of psoriasis, we stimulated HaCaT keratinocytes with M5, as previously described 27. M5 represents a combination of TNF-α, oncostatin M, IL-1α, IL-22, and IL-17A (PeproTech, Cranbury, NJ, USA;10 ng/mL for each cytokine). In brief, HaCaT cells (1.0 × 10^5^/well) were added to 96-well plates and cultured for 1 d. Subsequently, fresh serum-free medium supplemented with M5 was introduced into 96-well plates, and the cells were further cultured for an additional 1 d, in the presence of montelukast.

### Cell viability and proliferation of HaCaT keratinocytes

Cell viability was determined using the Cell Counting Kit-8 assay (CCK-8; KGA317; Nanjing KeyGen Biotech Co., Ltd., Nanjing, China). HaCaT keratinocytes were seeded in 96-well plates and incubated for 1 d. Cells with a density ranging from 50-60% were treated with montelukast for 24-48 h. Following the respective treatment periods, each well received 10 μl of CCK-8 and further cultured for 2 h at 37 °C with 5% CO_2_. The optical density was measured at 450 nm using a BioTek SYNERGY H1 plate reader (Agilent) and were found to be proportional to the number of live cells.

For the cell proliferation assays, HaCaT keratinocytes were incubated with montelukast and M5 cytokines. Subsequently, the CCK-8 assay was performed as previously described. Furthermore, 2 × 10^5^ HaCaT cells stimulated with M5 cytokines and labeled with carboxyfluorescein succinimidyl ester (CFSE, Invitrogen, 65-0850-85) underwent the specified montelukast treatment over a 2-d period.

### Real-time PCR

Total mRNA was extracted from HaCaT cells, murine and human skin samples, and mouse spleen cells using the TRIZOL kit (R401-01-AA, Vazyme biotech co., ltd.). The extracted RNA was used in the RT SuperMix (R323-01, Vazyme biotech co., ltd) for reverse transcription following the manufacturer's instructions for cDNA synthesis. A Roche LightCycler 480 II instrument (Roche, Mannheim, Germany) was used to perform a quantitative reverse transcription polymerase chain reaction (qPCR) using the SYBR method (Q711-02, Vazyme biotech co., ltd). Primers from Tsingke Biotech (Beijing, China) were used for the PCR reaction (Table S2). The internal reference was β-actin or GAPDH, and the relative level was measured using the 2^-ΔΔCt^ method.

### Flow cytometry

#### Immune cell detection using flow cytometry in irritated epidermis

To create the dermis dissociation buffer, the dilutions of stock solutions were prepared which included DNase I (10104159001, Roche) diluted to a concentration of 100 μg/mL, Collagenase P (11213857001, Roche) diluted to a concentration of 1 mg/mL, and HAase (abs47014926, Absin, Shanghai, China) diluted to concentration of 10 μg/mL. DMEM/high glucose (SH30022, Hyclone, Marlborough, MA, USA) was used here without antibiotics or fetal bovine serum. We prepared this buffer on the same day as of the sample processing and maintain it at a pre-chilled temperature of 4 °C. For skin digestion, 4.5 mL of this dermis dissociation buffer was used for each back skin sample. Single-cell suspensions were obtained by centrifuging the samples at 500 g for 5 min.

Roughly 2.0 × 10^6^ cells underwent a treatment phase involving mouse Fc-R blocking (553141, BD Biosciences, San Diego, CA, USA) for 10-15 min, at 20-25 °C. After this, surface staining was performed using specific marker antibodies for 45 min at 4 °C and avoiding exposure to light. Subsequently, the cells were fixed and permeabilized before intracellular labeling with Transcription Factor Buffer (562574, BD Biosciences). The cells were stained with specific fluorescence antibodies for 90 min at 4 °C. Flow cytometry data were analyzed using the FlowJo 10.8.1 software (https://fjinstallers.s3.amazonaws.com/FlowJo/FlowJo-Win64-10.8.1.exe). The flow cytometry reagents are as follows: Zombie Aqua™ Fixable Viability Kit (423102, BioLegend), APC-Cy™7 Rat Anti-Mouse CD3 Molecular Complex (560590, BD Biosciences), PE anti-mouse CD3 Antibody (100206, BioLegend), Alexa Fluor® 700 anti-mouse CD4 Antibody (100430, BioLegend), Brilliant Violet 605™ anti-mouse/human CD45R/B220 Antibody (103244, BioLegend), Brilliant Violet 785™ anti-mouse CD25 Antibody (102051, BioLegend), Brilliant Violet 605™ anti-mouse CD69 Antibody (104530, BioLegend), Brilliant Violet 421™ anti-mouse CD103 Antibody (121422, BioLegend), BCL6 Monoclonal Antibody (BCL-DWN), APC (17-5453-82, eBioscience, San Diego, CA, USA), CD278 (ICOS) Monoclonal Antibody (7E.17G9) (16-9942-85, eBioscience, San Diego, CA, USA), PE-Cyanine7 (25-9942-82, eBioscience), PE/Dazzle™ 594 anti-T-bet Antibody (644828, BioLegend), PE Mouse anti-Mouse RORγt (562607, BD Biosciences), Alexa Fluor® 488 anti-mouse/rat/human FOXP3 Antibody (320012, BioLegend), APC anti-mouse/human CD11b Antibody (101212, BioLegend), PerCP/Cyanine5.5 anti-mouse CD11c Antibody (117328, BioLegend), FITC Hamster Anti-Mouse γδ T-Cell Receptor (553177, BD Biosciences), PE/Cyanine7 anti-mouse I-A/I-E Antibody (107630, BioLegend), Brilliant Violet 785™ anti-mouse F4/80 Antibody (123141, BioLegend), and Brilliant Violet 650™ anti-mouse Ly-6G Antibody (127641, BioLegend). Flow cytometry was performed using a Cytek Northern Lights-CLC (NL-CLC V16B14R8, Cytek Biosciences, Fremont, CA, USA) and the gating strategy of skin lesions was shown in Figure S5.

### Spleens from IMQ-induced psoriasis model

Manual disruption, followed by filtration, was used to create cell suspensions from mouse spleen tissues. Then, 2.0 × 10^6^ cells were subjected to mouse Fc-R blocking (553141, BD Biosciences) for 10-15 min at 20-25 °C. Subsequently, surface staining was performed for 45 min at 4 °C under dark conditions using specific marker antibodies. The flow cytometry reagents used were: PerCP/Cyanine5.5 anti-mouse CD4 Antibody (116012, BioLegend), APC anti-mouse IFN-γ Antibody (505810, BioLegend), FITC anti-mouse CD3 Antibody (100204, BioLegend), Brilliant Violet 421™ anti-mouse IL-4 Antibody (504120, BioLegend), PE/Cyanine7 anti-mouse IL-17A Antibody (506922, BioLegend), PE Rat anti-Mouse Foxp3 (563101, BD Biosciences), APC-Cy™7 Rat Anti-Mouse CD3 Molecular Complex (560590, BD Biosciences), FITC anti-mouse/human CD44 Antibody (103022, BioLegend), Zombie Aqua™ Fixable Viability Kit (423102, BioLegend), Brilliant Violet 785™ anti-mouse CD25 Antibody (102051, BioLegend), PE anti-mouse CD279 (PD-1) Antibody (135206, BioLegend), APC Streptavidin (405207, BioLegend), Biotin Rat Anti-Mouse CD185 (CXCR5) (551960, BD Biosciences), Brilliant Violet 605™ anti-mouse/human CD45R/B220 Antibody (103244, BioLegend), Brilliant Violet 421™ anti-mouse, Alexa Fluor® 700 anti-mouse CD5 Antibody (100636, BioLegend), PE/Cyanine7 anti-mouse CD62L Antibody (104418, BioLegend), and CD1d (CD1.1, Ly-38) Antibody (123527, BioLegend). Flow cytometry was performed using Cytek Northern Lights-CLC (NL-CLC V16B14R8, Cytek Biosciences) and the gating strategy of spleens was shown in Figure S6.

### Cytometric bead array

Serum samples from IMQ-induced psoriasis-like mice were isolated to analyze cytokine levels using a kit of LEGENDplexTM Mouse Th Cytokine Panel (12-plex) (741044, BioLegend), following the manufacturer's instructions. Serum levels of various cytokines, including IFN-γ, TNF-α, IL-22, IL-17F, IL-17A, IL-10, IL-9, IL-6, IL-5, IL-4, and IL-2 were analyzed using flow cytometry on a flow cytometer (BD FACSVerseTM, BD Biosciences) and data were determined using the LEGENDplex software (https://www.biolegend.com/nl-be/immunoassays/legendplex).

### Human naïve CD4^+^ T-cell separation and Th17 polarization *in vitro*

To isolate human naïve CD4^+^ T cells, PBMCs were obtained from six healthy donors and subsequently purified using the CD4 Naïve T Cell Isolation Kit (480042, BioLegend). After purification, the CD4 naïve T cells were cultured in RPMI 1640 medium (21870076, Gibco) for 72 h under Th17 polarizing conditions, along with 2 μg/mL anti-CD3 (555329, BD Biosciences) and 1 μg/mL anti-CD28 (555725, BD Biosciences). The Th17 differentiation medium included 12.5 ng/mL IL-1β (200-01B-50, PeproTech), 25 ng/mL IL-6 (200-06-50, PeproTech), 5 ng/mL TGF-β1 (16-9243-85, Invitrogen), 10 µg/mL anti-IFN-γ (554698, BD Biosciences), and 10 µg/mL anti-IL-4 (16-7048-85, Invitrogen). Cultures were initiated in 12-well plates, with 1 × 10^6^ cells in each well, which were then incubated at 5% CO_2_ and 37 °C. Montelukast with different concentrations (0.2 μM, 2 µM, and 20 μM) was introduced into the culture, and for the control, dimethylsulfoxide (DMSO) was used. Following a 72-h incubation, all cells were harvested to assess Th17 cell differentiation. Additionally, the effect of montelukast on transcription factors associated with Th17 cell proliferation and differentiation was assessed using WB.

### CD4^+^ T-cell separation of CYSLTR1 KO mice

Spleen cells were isolated and purified from 8-week-old C57BL/6J mice (referred to as WT) and CYSLTR1 KO mice using the MojoSort™ Mouse CD4 T Cell Isolation Kit (480006, BioLegend). The cells were then seeded at a density of 5 × 10^5^ cells/well in 24-well plates. Activation was induced by adding 3 µg/mL plate-bound anti-CD3 (16-0031-86, Invitrogen) and 2 µg/mL anti-CD28 (16-0281-85, Invitrogen) antibodies for 4 d. After activation, various T cell subsets, including Th1, Th2, Th17, T follicular helper (Tfh), and T regulatory cells (Tregs), were assessed using flow cytometry through Cytek Northern Lights-CLC (NL-CLC V16B14R8, Cytek Biosciences). Furthermore, CD4^+^ T cells from WT and CYSLTR1 KO mice were also isolated and stimulated with anti-CD3/CD28 along with 100 nM LTD_4_ (LTD4; 20310, Cayman Chemical, Ann Arbor, MI, USA) or phosphate-buffered saline (PBS). After 4 d of culture, cells were harvested for WB analysis.

### Western blotting

Human CD4 naïve T cells were isolated and cultured in Th17 polarizing conditions for 4 d. CD4^+^ T cells from WT and CYSLTR1 KO mice were also isolated and stimulated with anti-CD3/CD28 along with 100 nM LTD_4_ or PBS. Cells were lysed used RIPA buffer (P0013K, Beyotime Biotechnology). Furthermore, spleen cells from CYSLTR1 KO mice that were subjected to IMQ modeling were extracted to assess the effect of CYSLTR1 KO on NF-κB signaling.

Protein samples were loaded onto polyvinylidene difluoride membranes after sodium dodecyl sulfate-polyacrylamide gel electrophoresis (SDS-PAGE; SLE009 Smart PAGE Precast Protein Gel, Smart-Life Sciences, Changzhou, China). Specific antibodies were used in WB to target the following proteins which included: 1:1000 NF-κB p-p65 (ser536) (3033, Cell Signaling Technology (CST), Danvers, MA, USA), 1:1000 NF-κB p65 (AF5006, Affinity Biosciences, Cincinnati, OH, USA), 1:1000 IKB alpha (T55026, Abmart, Shanghai, China), 1:1000 p-IKB alpha (Ser32/36) (TP56280, Abmart), 1:1000 STAT3 (AF6294, Affinity Biosciences), 1:1000 Phospho-Stat3 (Tyr705) (3357, CST), 1:1000 RORG (DF3196, Affinity Biosciences), and 1:2000 β-actin (4970S, CST). Proteins extracted from psoriatic lesions induced by IMQ were used for western blot analysis of CYSLTR1 (1:1000, PH6465, Abmart). The blots were visualized using a Tanon 5200 microscope (Tanon, Shanghai, China) and quantified using ImageJ version 1.8.

### Statistical analysis

Statistical analyses were performed on GraphPad Prism 9.5.0. Data are presented as mean ± standard error of the mean, assuming homogeneity and normal distribution of variances among groups. Two-tailed unpaired student's t-tests were used for comparisons between two groups. In cases involving multiple groups, one-way analysis of variance (ANOVA) was performed before appropriate post-hoc tests. When the assumptions of equal variance or normal distribution were not met, the non-parametric Mann-Whitney U test, a two-tailed analysis, was utilized. To maintain rigor and minimize potential sources of bias, the researchers remained blinded to group assignments throughout the entire course of the investigation.

## Results

### CYSLTR1 level was elevated in IMQ-induced psoriasis-like skin lesions and in patients with psoriasis

To assess the potential association between the expression of CYSLTR1 and psoriasis state, we measured CYSLTR1 levels in skin tissues obtained from both an IMQ-induced psoriasis-like murine model and normal BALB/c mice. Skin lesions from patients with psoriasis and healthy controls with pigmented nevi were collected and subjected to IHC and qPCR screening. We observed elevated expression of CYSLTR1 in IMQ-induced skin lesions (Figure 1B). Compared to healthy controls, higher *CYSLTR1* mRNA levels were observed in skin lesions (Figure 1C). IHC results showed a larger positively stained area for CYSLTR1 in both murine psoriasis model and patients (Figure 1D), and the cell counts of CD11c^+^ CYSLTR1^+^ cells and CD3^+^ CYSLTR1^+^ cells were significantly higher in the skin lesions of patients with psoriasis (Figure 1E). These findings suggest that CYSLTR1 is involved in the pathogenesis of psoriasis.

### Psoriasis phenotypes in mice were improved following topical and systemic administration of montelukast

**Figure 2:**
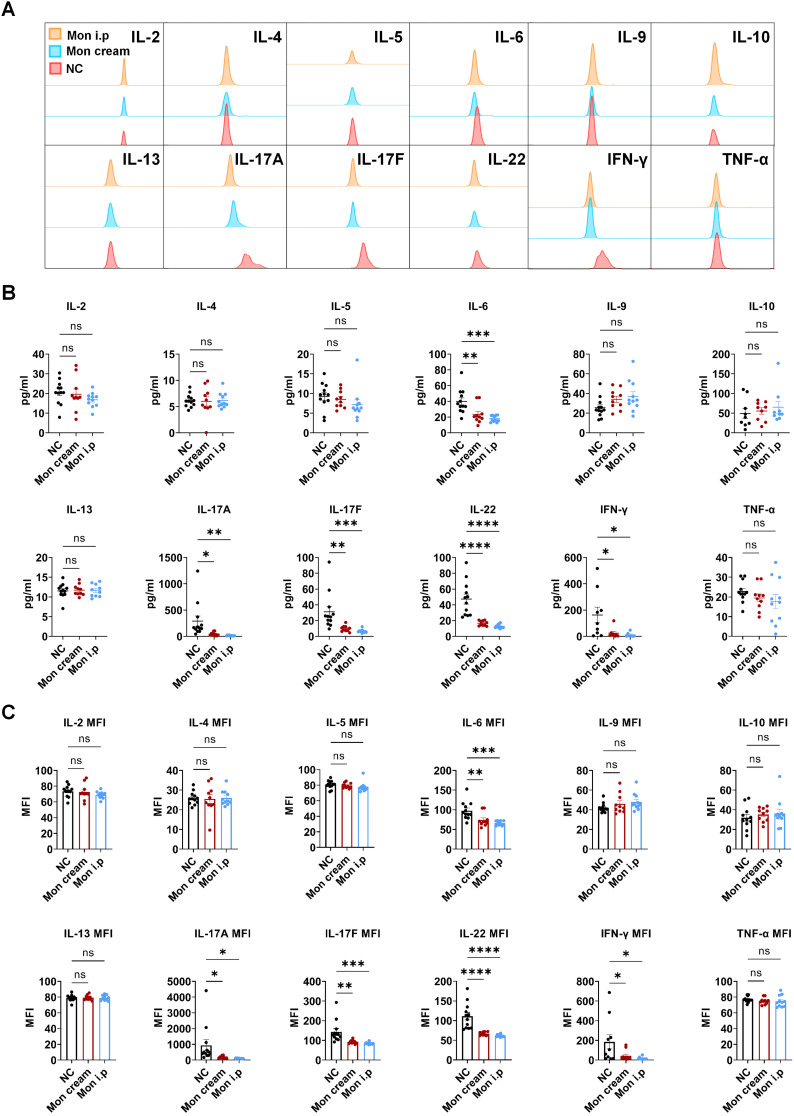
**Montelukast inhibits the production of serum inflammatory cytokines in the IMQ-induced psoriasis-like murine models. (A)** Representative flow cytometry diagrams and statistical analysis of the **(B)** concentrations and **(C)** mean fluorescence intensity (MFI) of the serum cytokines. n = 11 in IMQ + cream base (negative control: NC) group; n = 10 in IMQ + montelukast group; n = 11 in montelukast intraperitoneal (i. p) injection group. Horizontal bars represent the mean ± SEM, *p < 0.05, **p < 0.01, ***p < 0.001, ****p < 0.0001.

The elevated expression of CYSLTR1 in both the skin lesions of patients with psoriasis and IMQ-induced psoriasis implies the potential involvement of CYSLTR1 in the progression of psoriasis. Therefore, we evaluated the potential therapeutic effects of CYSLTR1 blockade in the progression of psoriasis. Treatment with montelukast sodium cream and an intraperitoneal injection of montelukast reduced the PASI scores and delayed the disease progression, as evidenced by improvements in skin erythema, scaling, and thickness in the IMQ-induced psoriasis murine model (Figure 1F). Furthermore, montelukast resulted in reduced levels of inflammatory cytokines, including IFN-γ, IL-22, IL-17A/F, and IL-6, by the two modes of administration (Figure 2A-C).

### Montelukast ameliorated the levels of inflammatory cells and psoriasis-related genes in the skin lesions

**Figure 3:**
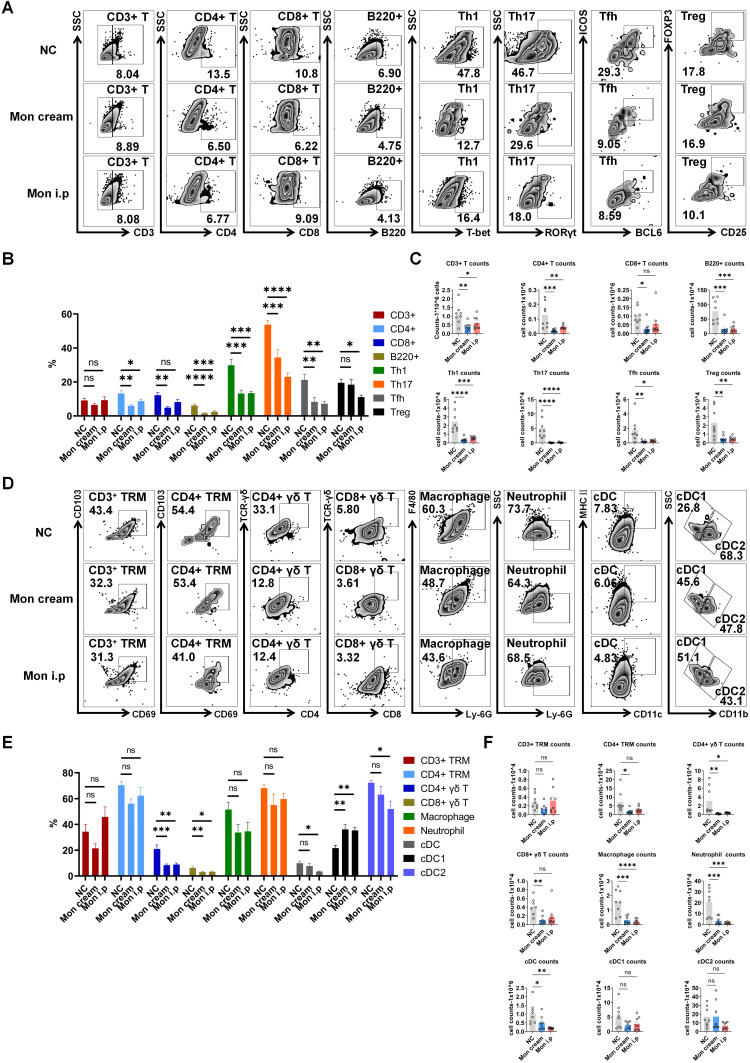
** Montelukast reduces the levels of inflammatory cells in the epidermis of the psoriasis-like mice. (A)** Representative flow cytometry diagrams and statistical analysis of the **(B)** frequencies and **(C)** numbers of T-cell subtypes (CD3^+^ T, CD4^+^ T, CD8^+^ T, Th1, Th17, Tfh, and Treg cells) and B220^+^ cells in the epidermis. **(D)** Representative flow cytometry diagrams and statistical analysis of the **(E)** frequencies and **(F)** numbers of CD3^+^/CD4^+^ TRM, CD4^+^/CD8^+^ γδ T, macrophage, neutrophil, cDC, cDC1 and cDC2 cells in the epidermis. n = 8 in IMQ + cream base (negative control: NC) group; n = 8 in IMQ + montelukast sodium cream group; n = 8 in IMQ + montelukast i. p group. Skin lesions of 8 animals were randomly selected from each group for flow cytometry analysis. Horizontal bars represent the mean ± SEM. *p < 0.05, **p < 0.01, ***p < 0.001, ****p < 0.0001.

To explore the anti-psoriatic effects of montelukast in the IMQ-induced model, we performed flow cytometry to evaluate alterations in immune cell populations and qPCR to investigate the mRNA expression of psoriasis-related genes within skin lesions. Following treatment with montelukast cream and solution, we observed significantly reduced frequencies and numbers of inflammatory cells, such as B220^+^ B cells, CD4/CD8+ T cells, Th1, Th17, and Tfh cells (Figure 3A-C), as well as CD4/CD8^+^ γδ T cells and CD4^+^ tissue-resident memory T cells (TRM) (Figure 3D-F) (*p* < 0.05). Moreover, montelukast suppressed the expression of various cytokines (IL-12A, IL-17A/F, and IL-23), chemokines (CCL2), and genes associated with keratinocyte proliferation (*KI67*), and autoimmunity (*LL37*) (Figure S2). For an exhaustive catalog of the examined mRNA expressions, please refer to Supplementary Table S2.

### Montelukast regulated the differentiation of T subtypes in IMQ-induced psoriasis-like mice

**Figure 4:**
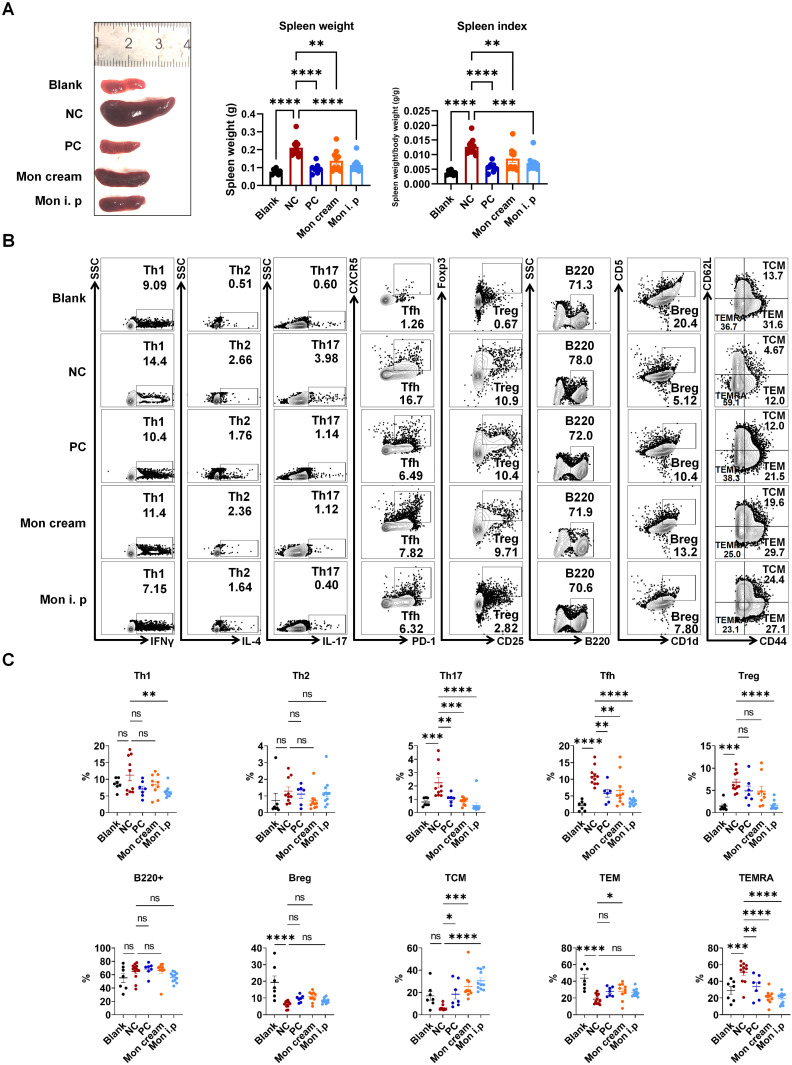
** Regulation of montelukast on splenic T and B cells in the IMQ-induced psoriasis murine model. (A)** Spleen weight and spleen index of the psoriasis-like mice. **(B)** Representative flow cytometry diagrams and **(C)** statistical analysis of the T cell-subtypes (Th1, Th2, Th17, Tfh, Treg cells, and CD4^+^ TCM/TEM/TEMRA) and B cell-subtypes (B220^+^ B, Breg cells) in the spleens. n = 7 in Blank group; n = 11 in IMQ + cream base (negative control: NC) group; n = 7 in IMQ + HMS (positive control: PC) group; n = 10 in IMQ + montelukast group, n = 11 in IMQ + montelukast i. p group. Horizontal bars represent the mean ± SEM, *p < 0.05, **p < 0.01, ***p < 0.001, ****p < 0.0001.

To gain deeper insights regarding the influence of montelukast on distinct immune cell subpopulations, we performed flow cytometry using splenocytes isolated from an IMQ-induced psoriasis-like model. The results indicated that treatment with montelukast sodium cream and solution led to a reduction in spleen weight and spleen index in IMQ-treated mice compared with those in the NC group (Figure 4A). Montelukast induced an essential effect on the number and frequency of T subsets, contributing to a reduction in Th17, Tfh, CD4^+^ central memory T cells (TCM), and CD4^+^ terminally differentiated effector memory CD45RA^+^ T cells (TEMRA). Interestingly, there were no significant differences in the frequencies of B220^+^ B cells or regulatory B cells (Bregs) between the montelukast-treated and control groups. Furthermore, in montelukast solution-treated mice, the fraction of Th1 decreased (Figure 4B, C).

### Montelukast regulated NF-κB signaling in IMQ-induced psoriasis-like skin lesions

**Figure 5:**
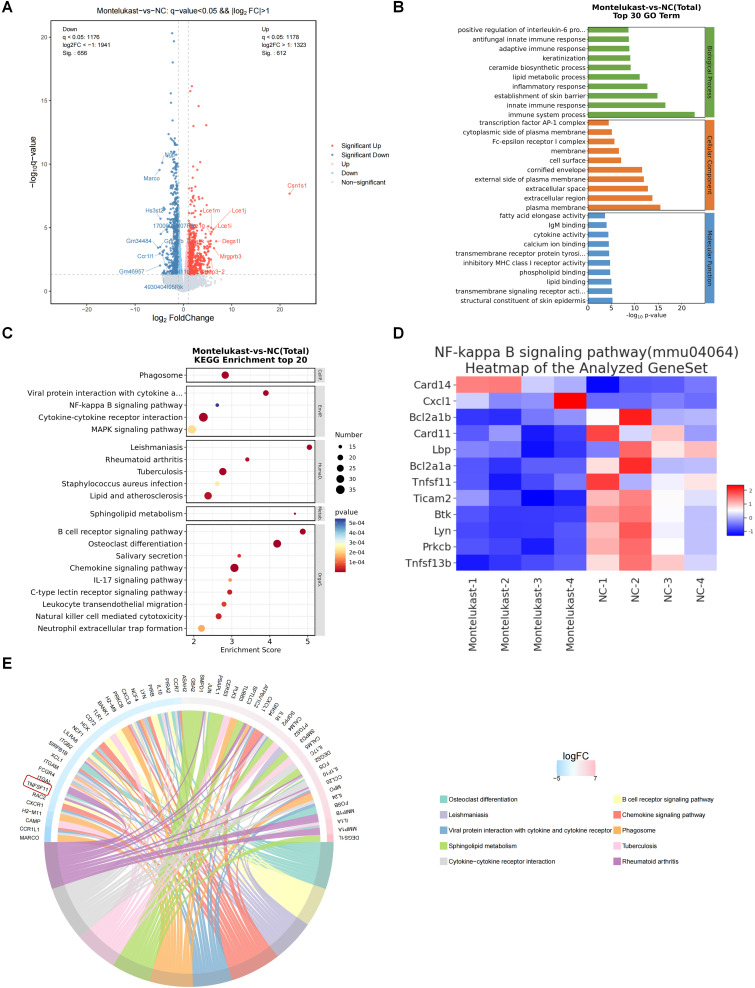
** RNA-Sequencing analysis of montelukast cream-treated mice. (A)** Volcano plot of differentially expressed genes (DEGs).** (B)** GO and** (C)** KEGG enrichment analysis of DEGs. **(D)** Clustering heatmap of the genes associated with the NF-κB signaling pathway. **(E)** A radar map of the top down and up-regulated 30 genes (up: red, down: blue) (NC = 4, montelukast cream = 4).

To delineate the features of the murine psoriasis model following treatment with montelukast cream, we used RNA-seq to elucidate the comprehensive transcriptomic profile of skin lesions isolated from the IMQ-induced psoriasis models. Among the 1268 DEGs, including 656 downregulated and 612 upregulated genes (Figure 5A), we performed GO enrichment (Figure 5B) and KEGG pathway analyses (Figure 5C). The outcomes underscored the engagement of these genes in inflammation-related signaling pathways, including the NF-κB pathway and IL-17 pathway. Following the application of montelukast cream, there was a significant suppression of NF-κB signaling-related genes (Figure 5D) compared to the control group. A radar map depicting the top 30 upregulated (depicted in red) and downregulated (depicted in blue) genes revealed *TNFSF11*, a gene associated with NF-κB signaling, among the top ten down-regulated genes (Figure 5E).

### Montelukast suppressed the proliferation of keratinocytes

**Figure 6:**
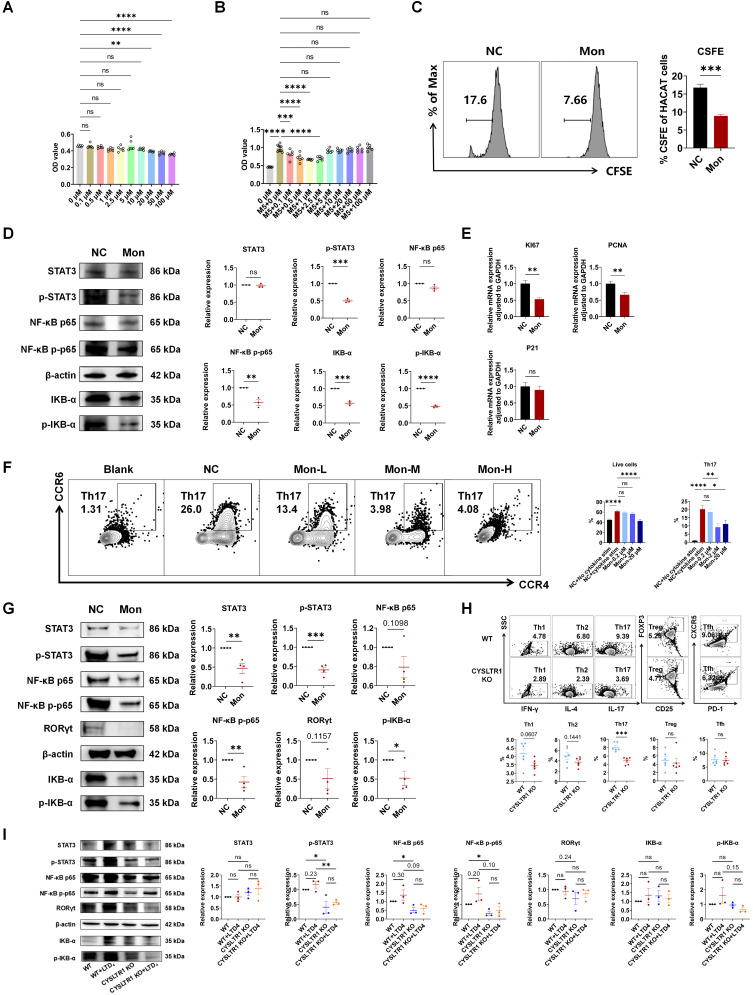
** Effect of Montelukast on HaCaT keratinocyte and Th17 cells.** OD value of CCK8 of montelukast treated HaCat cells **(A)** without M5 or **(B)** with M5 stimulation. **(C)** CFSE analysis of HaCat keratinocytes**. (D)** Montelukast suppressed the NF-κB signaling pathway in HaCat cells. **(E)** mRNA levels of genes associated with keratinocyte proliferation in M5-stimulated HaCaT keratinocytes.** (F)** Representative flow cytometry diagrams and statistical analysis of the frequencies of human naïve CD4^+^ T cells in Th17 polarization conditions. **(G)** Montelukast suppressed the NF-κB signaling pathway in human Th17.** (H)** Effect of CYSLTR1 knockout on mouse CD4^+^ T cells. **(I)** Effect of CYSLTR1 knockout and LTD_4_ on NF-κB signaling pathway in mouse CD4^+^ T cells. Horizontal bars represent the mean ± SEM, *p < 0.05, **p < 0.01, ***p < 0.001, ****p < 0.0001.

Aberrant keratinocyte hyperproliferation is a pivotal feature in the pathogenesis of psoriasis and addressing this aberration holds promise for significantly alleviating psoriasis 2. The CCK-8 assay results demonstrated that montelukast exhibited no cytotoxicity towards HaCaT cells at concentrations equal to or less than 10 μM (Figure 6A). To assess the antiproliferative effects of montelukast, we established an inflammatory keratinocyte model by stimulating HaCaT cells with M5. Following treatment with montelukast at 2.5 μM concentration for 48 h, montelukast exhibited notably enhanced inhibitory effects on keratinocyte growth when compared to the DMSO control through CCK-8 assay and CFSE assay (Figure 6B, C). Montelukast's inhibitory impact on HaCAT cell proliferation may be attributed to its modulation of the NF-κB signaling pathway (p-STAT3, NF-κB p-p65, and IKB-α/p-IKB-α) (Figure 6D). Furthermore, we examined the mRNA levels of genes related to proliferation (*KI67* and *PCNA*) (Figure 6E), angiogenesis (*TGF-α*, *VEGF*, and *HIF-1α*), keratogenesis (*KRT16*, *KRT17*, and *cyclinE1*), and autoimmunity (*S100A12*, *S100A15*, *hBD*-*2*, and *hBD*-*3*), and various pro-inflammatory cytokines (*IL-17A*, *IL-17F*, and *IL-6*), chemokines (*CXCL2* and *CXCL10*), in M5-stimulated HaCaT cells treated with montelukast (Figure S3).

### Blocking or knocking out CYSLTR1 inhibited Th17 cell differentiation by modulating NF-κB signaling

Montelukast exhibited a dose-dependent inhibition of the differentiation of human naïve CD4^+^ T cells into Th17 cells (*p* < 0.05) (Figure 6F), concomitant with the suppression of NF-κB signaling, including STAT3/p-STAT3, NF-κB p-p65, and p-IKB-α (Figure 6G). CYSLTR1 deficiency significantly inhibited CD4^+^ T cell differentiation into Th17 cells (*p* < 0.05; Figure 6H). Moreover, the CYSLTR1 agonist, LTD_4_, induced activation of NF-κB signaling, whereas the genetic knockout of CYSLTR1 in CD4^+^ T cells profoundly suppressed NF-κB signaling (p-STAT3, NF-κB p-p65/p65) (Figure 6I). Down regulation of NF-κB signaling pathway was also found in the skin lesion and spleen of CYSLTR1 KO mice after IMQ application (Figure S4).

### Attenuated psoriasis symptoms in CYSLTR1 KO mice

**Figure 7:**
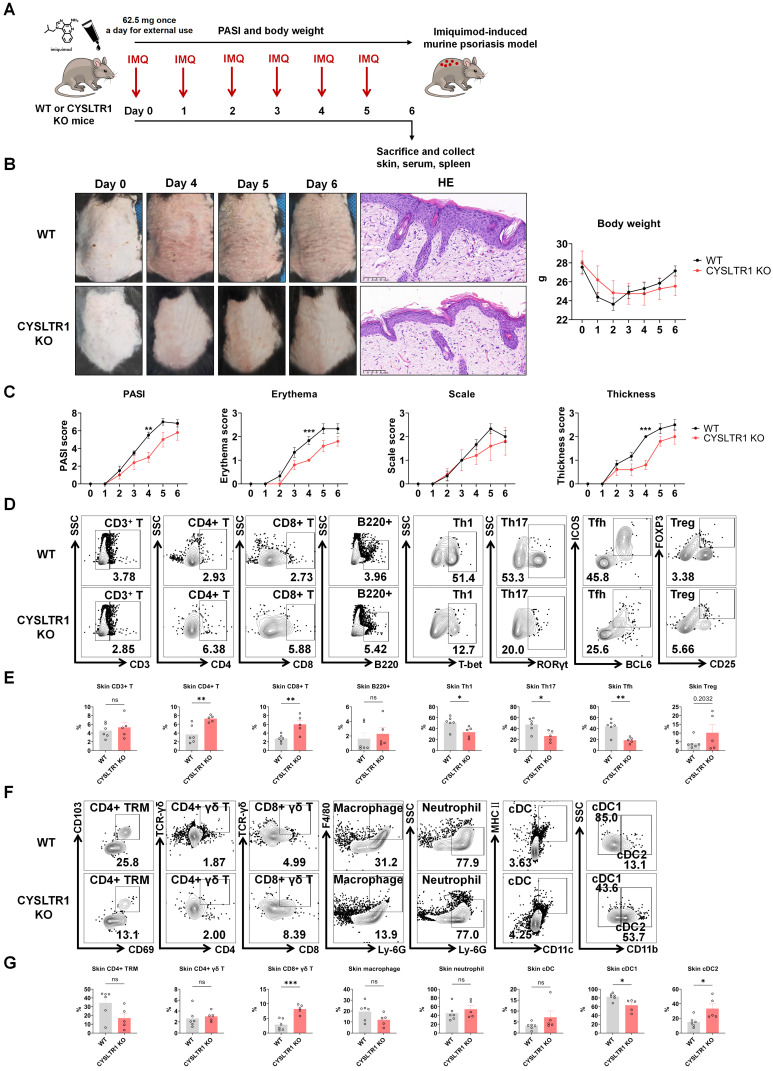
** Attenuated psoriasis symptoms in CYSLTR1 KO mice. (A)** A schematic of IMQ-induced psoriasis-like skin lesions in CYSLTR1 KO mice. **(B)** The skin lesions, histological features, and body weight of the psoriasis-like mice. **(C)** Changes in PASI scores, erythema, scale, and thickness scores. **(D & F)** Representative flow cytometry diagrams and **(E & G)** statistical analysis of the frequencies of immune cells in the epidermis. n = 6 in WT group; n = 5 in CYSLTR1 KO group. Horizontal bars represent the mean ± SEM, *p < 0.05, **p < 0.01, ***p < 0.001, ****p < 0.0001.

**Figure 8:**
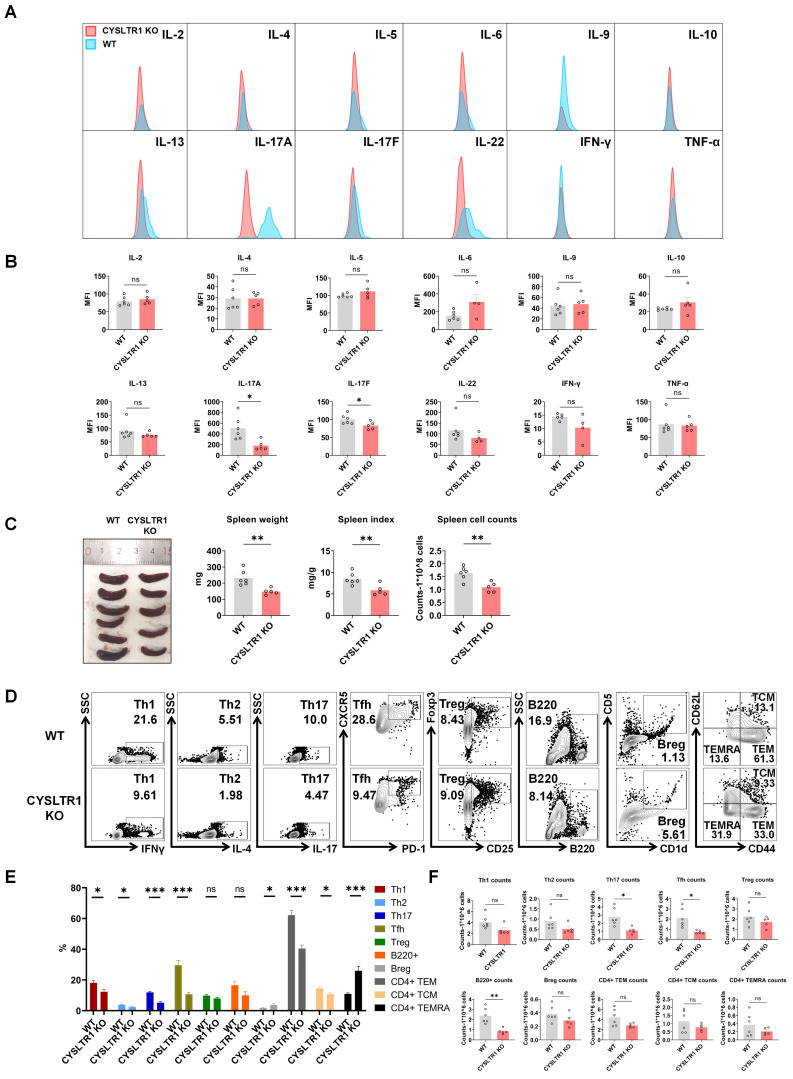
** Reduced serum inflammatory cytokines and splenic Th17 cells in CYSLTR1 KO mice. (A)** Representative flow cytometry diagrams and statistical analysis of **(B)** mean fluorescence intensity (MFI) of the serum cytokines. **(C)** Spleen weight and spleen index of the psoriasis-like mice. **(D)** Representative flow cytometry diagrams and statistical analysis of the **(E)** proportions and **(F)** numbers of T cell-subtypes (Th1, Th2, Th17, Tfh, Treg cells, CD4^+^ TCM/TEM/TEMRA) and B cell-subtypes (B220^+^ B, Breg cells) in the spleens. n = 6 in WT group; n = 5 in CYSLTR1 KO group. Horizontal bars represent the mean ± SEM, *p < 0.05, **p < 0.01, ***p < 0.001, ****p < 0.0001.

As montelukast exhibits substantial therapeutic benefits in the IMQ-induced psoriasis model, we next elucidated the role of CYSLTR1 in psoriasis progression (Figure 7A). Notably, CYSLTR1 KO mice exhibited reduced PASI scores and delayed disease progression, as evidenced by improvements in skin erythema, scale, and thickness compared to WT mice (Figure 7B, C). Moreover, CYSLTR1 KO mice resulted in a lower levels of T cell subtypes (Th1, Th17, and Tfh) (Figure 7D, E) and cDC1 (Figure 7F, G) within the skin lesions and reduced concentrations of serum inflammatory cytokines (IL-17A/F) (Figure 8A, B). Furthermore, CYSLTR1 KO led to reductions in spleen weight and spleen index (*p* < 0.05) (Figure 8C), alterations in the frequencies and/or numbers of Th1, Th2, Th17, Tfh, B220, and CD4^+^ effector memory T Cell (TEM) /TCM/TEMRA cells (*p* < 0.05), and increased Breg cells within the spleen (*p* < 0.05) (Figure 8D-F).

## Discussion

Montelukast, a highly selective inhibitor of CYSLTR1, was developed and manufactured by Merck & Co. 28. It was approved by the China National Medical Products Administration in 1999 and is primarily used to treat asthma. Research has also suggested its potential to modulate the transfer of Th17 cells and inhibit IL-17A secretion, thus providing relief in conditions such as MS 16. Our study findings aligned with prior observations in patients with psoriasis, indicating elevated CYSLTR1 levels in the IMQ-induced psoriasis model and in patients with psoriasis 18, higher levels of CD11c^+^ CYSLTR1^+^ cells and CD3^+^ CYSLTR1^+^ cells were also observed in psoriatic skins, CD11c^+^ DCs and CD3^+^ T cells contribute substantially to the pathogenesis of psoriasis 29. Given the heightened expression of CYSLTR1 in psoriatic lesions and the capacity of montelukast to restrain Th17 and IL-17, montelukast holds promise as a therapeutic agent for managing psoriasis characterized by hyperactive Th17 cells. However, the precise effect of CYSLTR1 on psoriasis development and the underlying mechanisms of CYSLTR1-targeted psoriasis treatment remain unclear.

Our study involved the development of a montelukast sodium cream using a multifactor orthogonal test. We demonstrated that inhibiting CYSLTR1 using montelukast sodium cream and systemic administration of montelukast sodium solution delayed disease progression in an IMQ-induced psoriasis murine model. This delay was evident, as observed in the reduction of PASI scores, and decreased infiltration of inflammatory cells and the thickness of the stratum spinosum.

Additionally, montelukast treatment resulted in lower serum concentrations of pro-inflammatory cytokines, including IL-6, IL-17A, IL-17F, IL-22, and IFN-γ. Montelukast also had a notable impact on specific T-cell subsets, reducing Th1, Th17, Tfh, CD4^+^ TRM, and CD4^+^ γδ T cells. Moreover, both topical and intraperitoneal administration of montelukast suppressed the mRNA levels of various cytokines (*IL-12A*, *IL-17A/F*, and *IL-23*), markers of keratinocyte proliferation (*KI67*, *PCNA*) 30, and genes associated with autoimmunity (*LL37*) 31 in skin lesions and played a role in regulating disease progression in the context of psoriasis 32. In addition, a reduced frequency of splenic Th17 and Tfh cells was observed after montelukast treatment. In summary, both the topical and systemic administration of montelukast showed positive therapeutic effects on psoriasis.

To elucidate the characteristics of the murine psoriasis model after treatment with montelukast cream, we used RNA-seq to reveal the comprehensive transcriptomic profile of skin lesions in IMQ-induced psoriasis models. Following montelukast cream application, significant suppression of NF-κB signaling-related genes was evident when compared to the control group, with *TNFSF11 (RANKL)*, a gene linked to NF-κB signaling 33, among the top ten down-regulated genes. RNA-seq findings suggested that montelukast might mitigate psoriasis progression by inhibiting the NF-κB signaling pathway.

*In vitro*, montelukast demonstrated an inhibitory effect on M5-induced overexpression of factors associated with hyperproliferation (KI67, PCNA), inflammatory cytokines (IL-17A, IL-17F 34, IL-6), angiogenesis (TGF-α 35, VEGF 36, and HIF-1α 37), keratogenesis (KRT16 38, KRT17 39, and cyclin E1 40), and autoimmunity (S100A12 41, S100A15 42, hBD-2 43, and hBD-3 44) in HaCaT keratinocytes. The inhibitory effect of montelukast on HaCAT cell proliferation may be attributed to the modulation of the NF-κB signaling pathway in keratinocytes. Activation of NF-κB is a key factor in the development of psoriasis 45. The NF-κB signaling pathway establishes links between altered keratinocyte and immune cell states, exerting influence over cellular proliferation, differentiation, apoptosis, chemokine, and cytokine production 46. These findings suggest the potential therapeutic outcomes of montelukast in psoriasis by modulating various key factors involved in the disease pathogenesis, including NF-κB signaling.

In addition, multiple studies have underscored the significant contributions of Th1, Th17, Tfh, CD4^+^ TRM, and CD4^+^ γδT cells, along with their secreted cytokines, in the development of psoriasis 47-51. The pivotal roles of Th17 cells and IL-17A in psoriasis pathogenesis have been extensively recognized. Monoclonal antibodies designed to target IL-17A (Ixekizumab and Secukinumab) have exhibited remarkable clinical outcomes in psoriasis treatment 52, 53. Consequently, our findings regarding the substantial inhibitory effects of montelukast on IL-17A and Th17 cells in mouse models of psoriasis emphasized its considerable potential as a therapeutic intervention for psoriasis. Moreover, our *in vitro* experiments revealed that montelukast treatment impedes the differentiation of naïve human CD4^+^ T cells into Th17 cells. Concomitantly, it downregulated critical components of the NF-κB pathway, including p-STAT3/STAT3 and NF-κB p-p65/NF-κB p65. CYSLTR1 deficiency was associated with notable inhibition of CD4^+^ T cell differentiation into Th17 cells. Furthermore, the CYSLTR1 agonist, LTD_4_, demonstrated the induction of NF-κB signaling activation, while the knockout of CYSLTR1 in CD4^+^ T cells resulted in a pronounced suppression of NF-κB signaling. Notably, the NF-κB pathway is pivotal in governing the differentiation and proliferation of Th17 cells and the secretion of IL-17. This regulatory role significantly contributes to the initiation and progression of psoriasis 54-56.

Given the remarkable therapeutic outcomes of the CYSLTR1 antagonist montelukast in the IMQ-induced psoriasis model, our initial approach involved establishing a psoriasis model in CYSLTR1 KO mice to elucidate the role of CYSLTR1 in the progression of psoriasis. Our study demonstrated reduced PASI scores and a significant delay in disease progression in CYSLTR1 KO mice compared with their WT counterparts. This improvement was accompanied by a notable reduction in the proportion of T cell subtypes, including Th1, Th17, and Tfh cells, in both the skin lesions and spleens of CYSLTR1 KO mice. Moreover, we observed a higher proportion of Bregs in these CYSLTR1 KO mice, which is particularly intriguing. Notably, IL-10-producing Bregs have been reported to be impaired and inversely correlated with IL-17- and IFN-γ-producing T cells in patients with psoriasis 57. Elevated levels of Bregs decreased the number of innate and inflammatory T cells in psoriasis 58, 59. The NF-κB signaling pathway was markedly inhibited in the skin and spleen of CYSLTR1 KO mice. These results highlighted the role of CYSLTR1 in psoriasis.

These results provide compelling evidence that CYSLTR1 upregulation drives the initiation and progression of psoriasis. We demonstrated that psoriasis can be effectively managed by blocking CYSLTR1 using topical montelukast cream and systemic administration or through genetic knockout approaches, primarily by regulating the NF-κB pathway within Th17 cells. These mechanistic insights strengthen the proposition that CYSLTR1 holds substantial promise as a therapeutic target, not only for psoriasis but also for autoimmune diseases underpinned by the dysregulation of Th17 cells. Furthermore, the newly developed montelukast cream has skin-friendly properties characterized by minimal irritation potential. This establishes it as a cost-effective and clinically viable alternative to systemic delivery, further emphasizing its potential clinical utility.

## Supplementary Material

Supplementary materials and methods, figures, and tables.
