# Supplementary material for: CYSLTR1 antagonist inhibits Th17 cell differentiation by regulating the NF-κB signaling for the treatment of psoriasis
Source: Int J Biol Sci. 2024 Mar 25;20(6):2168–86. doi: 10.7150/ijbs.92514 (PMC11008267; doi:10.7150/ijbs.92514)
Supplement: Supplementary file 1 — Supplementary materials and methods, figures, and tables. [file ijbsv20p2168s1.pdf]

Supplementary Materials

CYSLTR1 antagonist inhibits Th17 cell differentiation by regulating the NF-κB signaling for the treatment of psoriasis

Materials and Methods

Montelukast sodium cream

Chemicals and reagents

Montelukast sodium was obtained from Shanghai Yuanye Bio-Technology Co., Ltd. (Shanghai, China). Octadecanoic acid and glycerol were obtained from Sinopharm Chemical Reagent Co., Ltd. (Shanghai, China). Pharmaceutical-grade glyceryl monostearate, Vaseline, triethanolamine, ethyl 4-hydroxybenzoate, and dimethyl sulfoxide (DMSO) were obtained from Shanghai Aladdin Biochemical Technology Co., Ltd. (Shanghai, China).

Preparation of montelukast sodium cream

To formulate montelukast sodium cream, we performed a multifactor orthogonal test to optimize the preparation procedure. We considered several factors, including triethanolamine content (factor A), glyceryl monostearate content (factor B), emulsification temperature (factor C), and emulsification time (factor D), as the variables for investigation. The optimization process involved evaluating various parameters related to cream preparation quality, including appearance, pH, cold resistance, heat resistance, and centrifugal stability (Table S3). Subsequently, we successfully developed an optimized montelukast sodium cream.

The cream formulation comprised two distinct phases, an aqueous and an oil phase. The ingredients in the aqueous phase included triethanolamine (5.2%), glycerol (6.3%), DMSO (3.5%), montelukast sodium (3%), ethyl 4-hydroxybenzoate (0.1%), and purified water. The following constituents were present in the oil phase: octadecanoic acid (10.5%), glyceryl monostearate (5.2%), and vaseline (4.2%). Montelukast sodium was initially dissolved in DMSO under sulfoxide by heating. Subsequently, the montelukast sodium solution was blended with other components of the aqueous phase. Finally, the cream formulation was prepared by adding purified water.

To create an oil-in-water (O/W) emulsion for cream preparation, the oil phase was introduced into the aqueous phase to create an oil-in-water emulsion for cream preparation. Initially, the oil

phase components were combined and heated to 80 ℃ until complete melting occurred. Subsequently, the heated oil phase was carefully incorporated into the aqueous phase, maintained at 80 °C. The mixture was continuously stirred for 15 min. Afterward, the mixture was allowed to cool down to 40 °C and continued to be stirred for an additional 15 min to achieve emulsification. The O/W cream containing 3% montelukast sodium was considered complete after the emulsion was cooled to 25 ℃. The same technique was used to create the vehicle cream without montelukast.

Drug transdermal test

Chemicals and reagents

Montelukast sodium (High Performance Liquid Chromatography [HPLC] ≥ 99%) for HPLC analysis was purchased from Shanghai Yuanye Bio-Technology Co., Ltd. (Shanghai, China). Phosphate-buffered saline (PBS) was purchased from ServiceBio Technology (Wuhan, China). Methanol, Acetonitrile, and trifluoroacetic acid were obtained from Merck (Darmstadt, Germany). Deionized water was purified using a Milli-Q water purification system (Millipore, Billerica, MA, USA).

***In vitro* permeation test (IVPT)**

Skin samples used for *in vitro* permeation test (IVPT) were obtained from normal BALB/c mice. On the day of the experiment, the skin (2 cm × 3 cm) was collected and the subcutaneous fat was removed after euthanasia. A LOGAN DHC-6TD transdermal diffusion instrument with an automated fraction collector was used for the IVPT experiments. The volume and permeation area of the diffusion cell were 12 mL and 1.767 cm^2. The receiver solution was phosphate-buffered saline (pH 7.4), and ultrasound was used for 30 min to remove bubbles. The speed of magnetic stirring was 600 r/min, and the temperature was maintained at 32 ± 0.2 ℃. The receiving cells were stirred, and the pretreated skin was fixed between the diffusion and receiving cells. The prepared montelukast sodium cream was taken and closely applied to the pretreated skin in a diffusion cell. Timing: 6 mL of the receiving solution was collected at time points of 5 min, 1 h, 2 h, 4 h, 6 h, 8 h, and 10 h. Fresh receiving solutions of the same volume were added simultaneously. The resulting receiver solution samples were analyzed by HPLC. The rate of montelukast sodium

permeation through the skin and the cumulative total permeation of montelukast sodium at different sampling time points were chosen as two key parameters to evaluate the skin permeation of montelukast sodium.

HPLC analysis of samples

The solvent of the resulting receiver solution samples was first removed using a Termovap Sample Concentrator, and then the samples were dissolved in methanol (1 mL). After passing it over 0.22 μM microporous filter membrane, the IVPT samples for 20 μl were injected and analyzed using a SHIMADZU LC-2050C HPLC system (Kyoto, Japan). A HPLC method was used to quantify the IVPT samples. BDS Hypersil™ C18 (2.4 μm, 4.6×100 mm) column was used to elute montelukast sodium. The mobile phase comprised 0.1% trifluoroacetic acid (A, 55%) and acetonitrile (B, 45%), with equal elution for 20 min. The flow rate was 1.0 ml/min. Montelukast sodium peak was detected at 220 nm.

Results

Determination of the level of investigation factors in the preparation of montelukast sodium cream

The role of emulsifiers in cream preparation is well established, with triethanolamine and glyceryl monostearate being common choices. Triethanolamine serves as both an emulsifier and an alkalizing agent, contributing to the formation of a uniform and stable cream for topical applications [1]. On the other hand, to make the cream base, glyceryl monostearate self-emulsifies and esterifies with stearic acid [2]. Consequently, the quantities of triethanolamine and glyceryl monostearate were identified as critical variables in formulating the montelukast sodium cream. Furthermore, the emulsification temperature and duration have been recognized as pivotal factors in the preparation process.

Optimization of the preparation process of 3% montelukast sodium creams by multi-factor orthogonal test

The montelukast sodium cream preparation process was optimized using an orthogonal experimental design. Specifically, we employed an L_9 (3^4) orthogonal design table for this

experiment, incorporating four factors, each with three levels. These four factors and their respective levels included triethanolamine content (Factor A): 4.0%, 4.6%, and 5.2%; glyceryl monostearate content (Factor B): 4.2%, 5.2%, and 6.2%; emulsification temperature (Factor C): 75 °C, 80 °C, and 85 °C; and emulsification time (Factor D): 15 min, 20 min, and 25 min (Table S4). The experimental results were analyzed using SPSS 19.0 (IBM Corp., Armonk, NY, USA).

As presented in Table S5, the R values for the four factors followed the order D > C > A > B. An analysis of the extreme differences revealed that the order of influence of the four factors on the experimental outcomes was D > C > A > B. The values K1', K2', and K3' denoted the average comprehensive scores associated with levels 1, 2, and 3 for each factor, respectively. The highest average comprehensive score for Factor A was 94.30, corresponding to Level 3. Regarding Factor B, the highest average comprehensive score was 94.07, which was attributed to Level 2. For Factor C, the highest average comprehensive score was 94.23 at level 2. Finally, the average comprehensive score for Factor D was 94.33, representing Level 1. In conclusion, the optimal preparation process for montelukast sodium cream was identified as A3B2C2D1, which corresponds to a triethanolamine content of 5.2%, glyceryl monostearate content of 5.2%, an emulsification temperature of 80 °C, and an emulsification time of 15 min. As shown in Table S6, all four factors significantly influenced the experimental outcomes. Consequently, A3B2C2D1 was established as the optimal protocol for the preparation of montelukast sodium cream.

In vitro permeation test of montelukast sodium cream

As presented in Figure S1A and B, montelukast sodium was detected at 14.742 min. The flux profile delineates the permeation rate of montelukast sodium through the skin at various sampling intervals, including 5 min, 1 h, 2 h, 4 h, 6 h, 8 h, and 10 h (Figure S1C). While the permeation rate of montelukast sodium remained constant at 0 from 5 min to 2 h, it subsequently increased to 38.4524, 66.2457, 184.5740, and 92.3390 ng/cm^2 from 4 h to 10 h, respectively. Figure S1D illustrates the cumulative total permeation of montelukast sodium at different time points, registering 0, 0, 0, 153.8094, 551.2837, 2027.8756, and 2951.2651 ng/cm^2, correspondingly. These observations suggest that montelukast sodium can permeate the skin following a 2-hour application of montelukast sodium cream. Furthermore, the rate of montelukast sodium permeation through the skin peaks at 8 h before gradually declining.

References

[1] S. Baptista, F. Freitas, Formulation of the Polysaccharide FucoPol into Novel Emulsified Creams with Improved Physicochemical Properties, Molecules 27(22) (2022).
[2] S.S. Hettiarachchi, Y. Perera, S.P. Dunuweera, A.N. Dunuweera, S. Rajapakse, R.M.G. Rajapakse, Comparison of Antibacterial Activity of Nanocurcumin with Bulk Curcumin, ACS Omega 7(50) (2022) 46494-46500.

Tables

Table S1. Clinical skin sample information of psoriasis patients and healthy controls from the Institute of Dermatology, Chinese Academy of Medical Sciences for IHC and qPCR screening.

No.	Gender	Age (years)	No.	Gender	Age (years)
Psoriasis1	Male	39	HC1	Female	24
Psoriasis2	Male	70	HC2	Female	26
Psoriasis3	Female	36	HC3	Male	31
Psoriasis4	Male	73	HC4	Female	23
Psoriasis5	Female	73	HC5	Male	35
Psoriasis6	Male	62	HC6	Male	9

Table S2. Primers for qPCR.

Species	Gene	Direction	Sequence (5'→3')
Mouse	*IL-1β*	Former	TGGACCTTCCAGGATGAGGACA
		Reverse	GTTCATCTCGGAGCCTGTAGTG
Mouse	*IL-6*	Former	CTGCAAGAGACTTCCATCCAG
		Reverse	AGTGGTATAGACAGGTCTGTTGG
Mouse	*IL-12A*	Former	CCCTTGCCCTCCTAAACCAC
		Reverse	CAGGTTTCGGGACTGGCTAA
Mouse	*IL-17A*	Former	TCCAGAAGGCCCTCAGACTA
		Reverse	CTCGACCCTGAAAGTGAAGG
Mouse	*IL-23*	Former	TCCTCCAGCCAGAGGATCACCC
		Reverse	AGAGTTGCTGCTCCGTGGGC
Mouse	*TNF-α*	Former	CCTGTAGCCCACGTCGTAG
		Reverse	GGGAGTAGACAAGGTACAACCC
Mouse	*IFN-γ*	Former	ATGAACGCTACACACTGCATC
		Reverse	CCATCCTTTTGCCAGTTCCTC
Mouse	*CCL2*	Former	CAGCCAGATGCAATCAATGCC
		Reverse	TGGAATCCTGAACCCACTTCT
Mouse	*CCL5*	Former	GCTGCTTTGCCTACCTCTCC
		Reverse	TCGAGTGACAAACACGACTGC
Mouse	*CCL20*	Former	GCCTCTCGTACATACAGACGC
		Reverse	CCAGTTCTGCTTTGGATCAGC
Mouse	*CXCL1*	Former	CTGGGATTCACCTCAAGAACATC
		Reverse	CAGGGTCAAGGCAAGCCTC
Mouse	*CXCL10*	Former	CCAAGTGCTGCCGTCATTTTC
		Reverse	GGCTCGCAGGGATGATTTCAA
Mouse	*LL37*	Former	GCTGTGGCGGTCACTATCAC
		Reverse	TGTCTAGGGACTGCTGGTTGA
Mouse	*S100A7*	Former	TGCTCTTGGATAGTGTGCCTC

		Reverse	GCTCTGTGATGTAGTATGGCTG
Mouse	*S100A8*	Former	AAATCACCATGCCCTCTACAAG
		Reverse	CCCACTTTTATCACCATCGCAA
Mouse	*S100A9*	Former	ATACTCTAGGAAGGAAGGACACC
		Reverse	TCCATGATGTCATTTATGAGGGC
Mouse	*β-actin*	Former	GTGACGTTGACATCCGTAAAGA
		Reverse	GCCGGACTCATCGTACTCC
Human	*IL-1β*	Former	ATGATGGCTTATTACAGTGGCAA
		Reverse	GTCGGAGATTCGTAGCTGGA
Human	*IL-6*	Former	ACTCACCTCTTCAGAACGAATTG
		Reverse	CCATCTTTGGAAGGTTCAGGTTG
Human	*IL-8*	Former	TTTTGCCAAGGAGTGCTAAAGA
		Reverse	AACCCTCTGCACCCAGTTTTC
Human	*IL-12A*	Former	ATGGCCCTGTGCCTTAGTAGT
		Reverse	AGCTTTGCATTCATGGTCTTGA
Human	*IL-12B*	Former	ACCCTGACCATCCAAGTCAAA
		Reverse	TTGGCCTCGCATCTTAGAAAG
Human	*IL-17A*	Former	TCCCACGAAATCCAGGATGC
		Reverse	GGATGTTCAGGTTGACCATCAC
Human	*IL-17F*	Former	GCTGTCGATATTGGGGCTTG
		Reverse	GGAAACGCGCTGGTTTTCAT
Human	*IL-18*	Former	TCTTCATTGACCAAGGAAATCGG
		Reverse	TCCGGGGTGCATTATCTCTAC
Human	*IL-20*	Former	ATGAAAGCCTCTAGTCTTGCCT
		Reverse	GCCCCGTATCTCAGAAAATCC
Human	*IL-21*	Former	TAGAGACAAACTGTGAGTGGTCA
		Reverse	GGGCATGTTAGTCTGTGTTTCTG
Human	*IL-22*	Former	GCTTGACAAGTCCAACTTCCA
		Reverse	GCTCACTCATACTGACTCCGT
Human	*IL-23*	Former	CTCAGGGACAACAGTCAGTTC
		Reverse	ACAGGGCTATCAGGGAGCA
Human	*IL-36A*	Former	GAACTCCACCTTCGAGTCTGT
		Reverse	CCCAAAGTCAGTAGTGTTGGC
Human	*TNF-α*	Former	CTCTTCTGCCTGCTGCACTTTG
		Reverse	ATGGGCTACAGGCTTGTCACTC
Human	*CXCL1*	Former	CTTGCCTCAATCCTGCATCC
		Reverse	CTCTGCAGCTGTGTCTCTCT
Human	*CXCL2*	Former	ACCAGAAGGAAGGAGGAAGC
		Reverse	CTCTGCAGCTGTGTCTCTCT
Human	*CXCL10*	Former	GTGGCATTCAAGGAGTACCTC
		Reverse	TGATGGCCTTCGATTCTGGATT
Human	*CCR6*	Former	CCTGACTTGCATTAGCATGGA
		Reverse	GCGGTAGTGTTCTGGATCGG
Human	*CCL2*	Former	CAGCCAGATGCAATCAATGCC

		Reverse	TGGAATCCTGAACCCACTTCT
Human	*CCL5*	Former	CCAGCAGTCGTCTTTGTCAC
		Reverse	CTCTGGGTTGGCACACACTT
Human	*CCL7*	Former	TGCTCAGCCAGTTGGGATTA
		Reverse	GCTACTGGTGGTCCTTCTGT
Human	*CCL8*	Former	TGGAGAGCTACACAAGAATCACC
		Reverse	TGGTCCAGATGCTTCATGGAA
Human	*CC13*	Former	CTCAACGTCCCATCTACTTGC
		Reverse	TCTTCAGGGTGTGAGCTTTCC
Human	*CCL20*	Former	TGCTGTACCAAGAGTTTGCTC
		Reverse	CGCACACAGACAACTTTTTCTTT
Human	*TGF-α*	Former	AGGTCCGAAAACACTGTGAGT
		Reverse	AGCAAGCGGTTCTTCCCTTC
Human	*VEGF*	Former	AGGGCAGAATCATCACGAAGT
		Reverse	AGGGTCTCGATTGGATGGCA
Human	*HIF-1α*	Former	GAACGTCGAAAAGAAAAGTCTCG
		Reverse	CCTTATCAAGATGCGAACTCACA
Human	*KI67*	Former	ACGCCTGGTTACTATCAAAAGG
		Reverse	CAGACCCATTTACTTGTGTTGGA
Human	*PCNA*	Former	CCTGCTGGGATATTAGCTCCA
		Reverse	CAGCGGTAGGTGTCGAAGC
Human	*P21*	Former	CTCTCCCGAAAAGCAGTCCC
		Reverse	GCGAGAACGAGCCAACCTT
Human	*S100A7*	Former	ACGTGATGACAAGATTGACAAGC
		Reverse	GCGAGGTAATTTGTGCCCTTT
Human	*S100A8*	Former	ATGCCGTCTACAGGGATGAC
		Reverse	ACTGAGGACACTCGGTCTCTA
Human	*S100A9*	Former	GGTCATAGAACACATCATGGAGG
		Reverse	GGCCTGGCTTATGGTGGTG
Human	*S100A12*	Former	AGCATCTGGAGGGAATTGTCA
		Reverse	GCAATGGCTACCAGGGATATGAA
Human	*S100A15*	Former	ACTCAAGCTGAGAGGTCCATAA
		Reverse	GCCATCACGTCCGGTGTATTT
Human	*LL37*	Former	GGCTGGTGAAGCGGTGTAT
		Reverse	TGGGTACAAGATTCCGCAAAAA
Human	*LCN2*	Former	CCACCTCAGACCTGATCCCA
		Reverse	CCCCTGGAATTGGTTGTCCTG
Human	*hBD-2*	Former	GGTGGTATAGGCGATCCTGTT
		Reverse	AGGGCAAAAGACTGGATGACA
Human	*hBD-3*	Former	TCCAGGTCATGGAGGAATCAT
		Reverse	CGAGCACTTGCCGATCTGT
Human	*KRT1*	Former	CTTTTCTGCTGTTTCCCAATGAA
		Reverse	GGAAAGAACAAAGCAGGGTCATAG
Human	*KRT6A*	Former	GACCTGGTGGAGGACTTCAA

		Reverse	CTTGGCTTGCAGTTCAACCT
Human	*KRT7*	Former	GTACCAGGAACTCATGAGC
		Reverse	TCATCACAGAGATATTCACGG
Human	*KRT10*	Former	TCCTACTTGGACAAAGTTCGGG
		Reverse	CCCCTGATGTGAGTTGCCA
Human	*KRT14*	Former	CACAGATCCCACTGGAAGAT
		Reverse	GATAATGAAGCTGTATTGATTGCC
Human	*KRT16*	Former	GACCGGCGGAGATGTGAAC
		Reverse	CTGCTCGTACTGGTCACGC
Human	*KRT17*	Former	GGTGGGTGGTGAGATCAATGT
		Reverse	CGCGGTTCAGTTCCTCTGTC
Human	*TGM1*	Former	GCACCACACAGACGAGTATGA
		Reverse	GGTGATGCGATCAGAGGATTC
Human	*CDK2*	Former	CCAGGAGTTACTTCTATGCCTGA
		Reverse	TTCATCCAGGGGAGGTACAAC
Human	*CDK4*	Former	ATGGCTACCTCTCGATATGAGC
		Reverse	CATTGGGGACTCTCACACTCT
Human	*Cyclin E1*	Former	AAGGAGCGGGACACCATGA
		Reverse	ACGGTCACGTTTGCCTTCC
Human	*CYSLTR1*	Former	AAGTCCGTGGTCATAACCTTGT
		Reverse	TCTGGGTACATAAGTCACGCT
Human	*GAPDH*	Former	GGAGCGAGATCCCTCCAAAAT
		Reverse	GGCTGTTGTCATACTTCTCATGG

Table S3. Evaluation criteria for montelukast sodium cream.

Evaluation basis	Evaluation index	Score	Grading
Appearance quality	Color and evenness	4 points	Uniform color, pure white or milky white
		2 points	Yellowish
		0 points	Uneven color with spots
	Texture	4 points	Smooth and delicate, without any particle feeling
		2 points	Slight particle feeling
		0 points	Large particle feeling
	Consistence	4 points	Scratches can be made and restored to its original state in a short period of time
		2 points	Unable to restore to its original state in a short period of time
		0 points	Relatively thin and flowable
	Spreadability	4 points	Easy to apply, not whitening
		2 points	Whitening
		0 points	Not easy to apply
	Cleanablity	4 points	Cleanable without using cleaning agents
		2 points	Cleanable with cleaning agents
		0 points	Not easy to clean
Acid-base property	pH	20 points	pH 5.5~7.5
		0 points	Other pH value
Stability	Centrifugal stability	20 points	No oil water stratification, demulsification, coarsening, and no bubble formation
		10 points	Mild phenomenon
		0 points	Obvious phenomenon
	Heat-resistant stability and cold-resistant stability	20 points	No oil water stratification, demulsification, coarsening, and no bubble formation, and the color is uniform without significant difference from the original product
		10 points	Mild phenomenon
		0 points	Obvious phenomenon

Table S4. Multi-factor orthogonal test design.

Levels	Factors			
	A (%)	B (%)	C (°C)	D (min)
1	4.0	4.2	75	15
2	4.6	5.2	80	20
3	5.2	6.2	85	25

Table S5. Scheme and results of L_9 (3^4) orthogonal experiments.

Test number	Factors				Comprehensive score (points)
	A (%)	B (%)	C (°C)	D (min)	
1	1	1	1	1	94.0
2	1	2	2	2	94.0
3	1	3	3	3	92.8
4	2	1	2	3	93.7
5	2	2	3	1	94.0
6	2	3	1	2	93.2
7	3	1	3	2	93.7
8	3	2	1	3	94.2
9	3	3	2	1	95.0
K_1	280.8	281.4	281.4	283.0	
K_2	280.9	282.2	282.7	280.9	
K_3	282.9	281.0	280.5	280.7	
K_1'	93.60	93.80	93.80	94.33	
K_2'	93.63	94.07	94.23	93.63	
K_3'	94.30	93.67	93.50	93.57	
R	2.1	1.2	2.2	2.3	

Table S6. Variance analysis for L_9(3^4) orthogonal test.

Factors	S.S.	df	M.S.	*P*
A	0.936	2	0.468	< 0.05
B	0.249	2	0.124	< 0.05
C	0.816	2	0.408	< 0.05
D	1.082	2	0.541	< 0.05

S.S., sum of squares; df, degree of freedom; M.S., mean of squares; A, the content of Triethanolamine (%); B, the content of Glyceryl monostearate (%); C, emulsification temperature (°C); D, emulsification time (min).

Figures

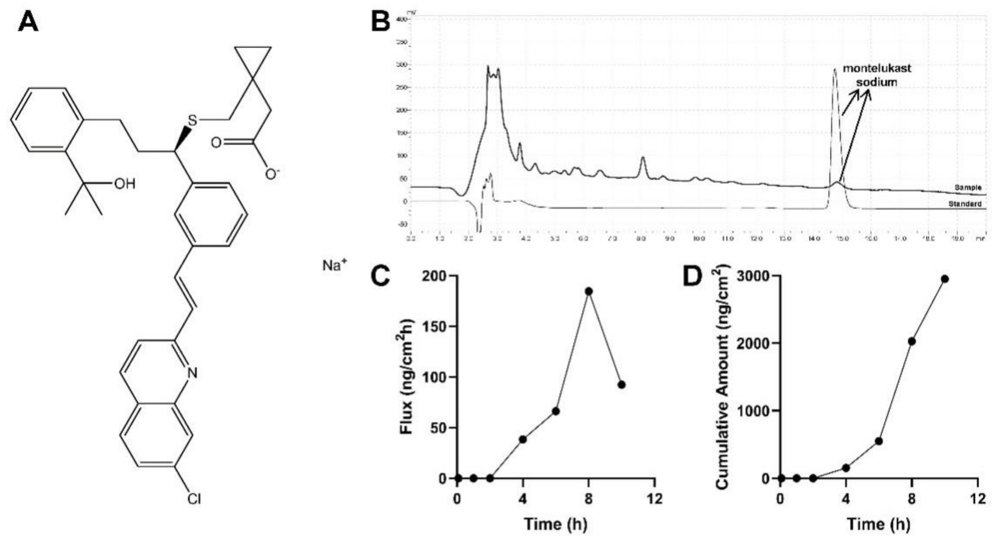

Figure S1. *In vitro* permeation test of montelukast sodium cream. **(A)** The chemical structure of montelukast sodium. **(B)** Representative HPLC chromatograms of the montelukast sodium standard solution and the resulting receiver solution sample. **(C)** The flux profile showing the rate of montelukast sodium permeating through the skin at different sampling time points, including 5 min, 1 h, 2 h, 4 h, 6 h, 8 h, and 10 h. **(D)** The cumulative total permeation of montelukast sodium at different sampling time points, including 5 min, 1 h, 2 h, 4 h, 6 h, 8 h, and 10 h.

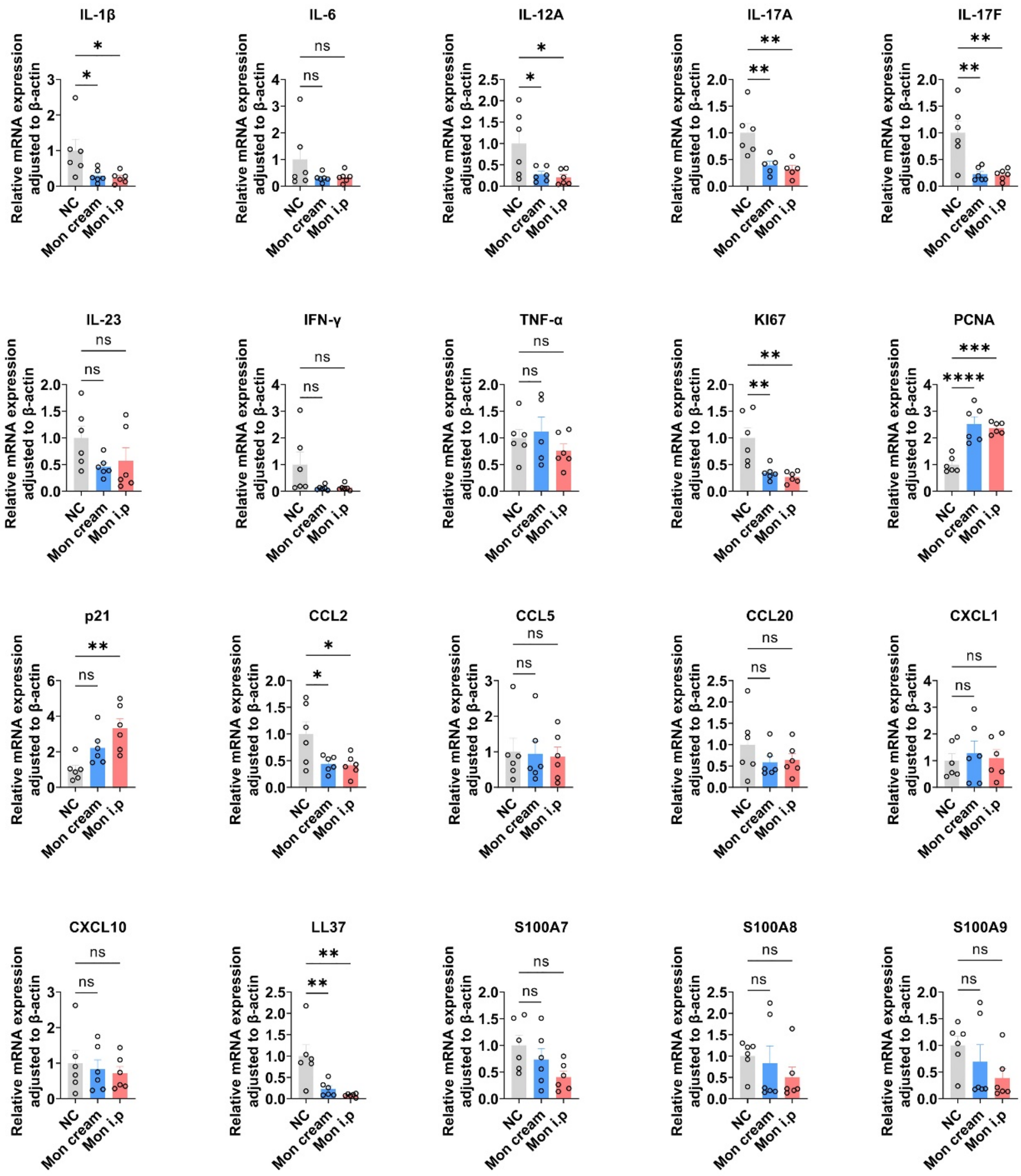

Figure S2. Skin mRNA levels of cytokines, chemokines, genes associated with keratinocyte proliferation and autoimmunity in IMQ-induced psoriasis-like mice. n = 6 in IMQ + cream base (negative control: NC) group; n = 6 in IMQ + montelukast cream group; n = 6 in IMQ + montelukast i. p group. 6 animals were randomly selected from each group for qPCR analysis. Horizontal bars represent the mean ± SEM. **p* < 0.05, ***p* < 0.01, ****p* < 0.001, *****p* < 0.0001.

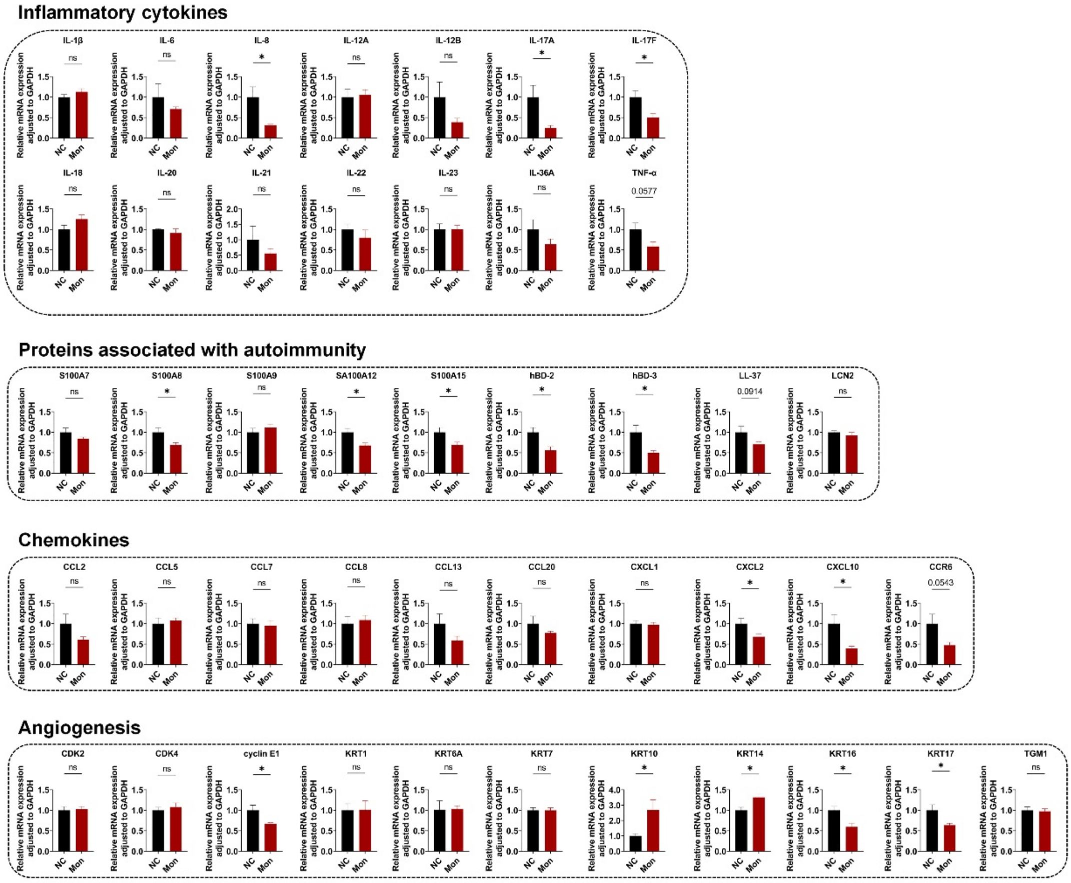

Figure S3. Montelukast's function in regulating mRNA levels of pro-inflammatory cytokines, and genes associated with keratogenesis of keratinocyte, as well as angiogenesis, autoimmunity, and chemokines in the skin lesions of IMQ-induced psoriasis-liked mice. Horizontal bars represent the mean ± SEM. *$p < 0.05$.

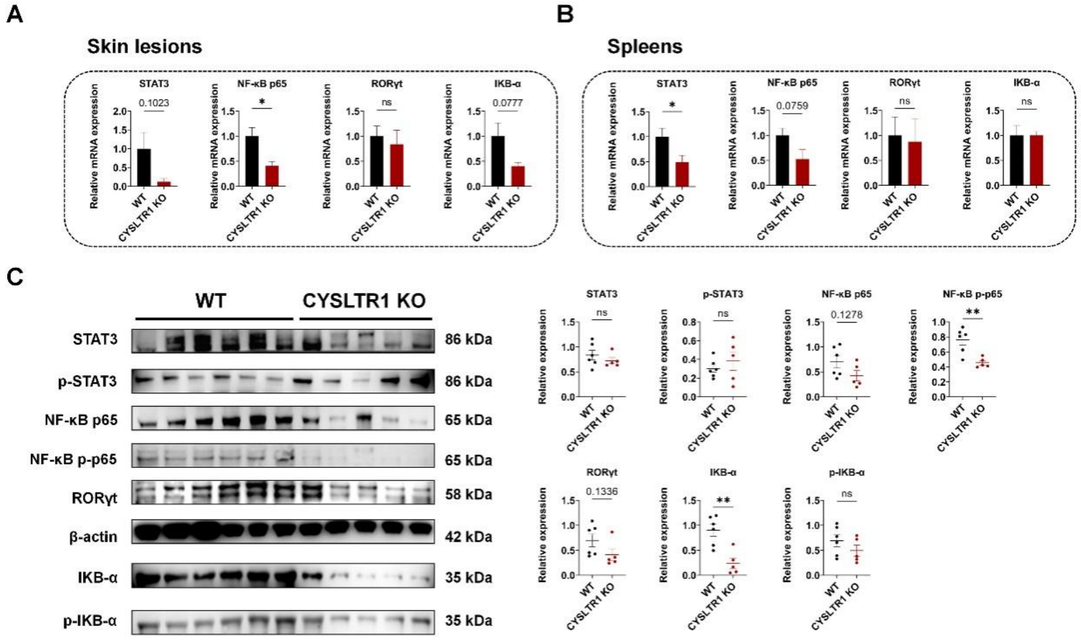

Figure S4. The effect of CYSLTR1 knockout on the NF-κB signaling pathway after IMQ application. qPCR screening of NF-κB signaling-related genes in the **(A)** skin lesions and **(B)** spleen tissues. **(C)** Western blotting of NF-κB signaling pathway in the spleens from WT and CYSLTR1 KO mice. Horizontal bars represent the mean ± SEM. *$p < 0.05$, **$p < 0.01$.

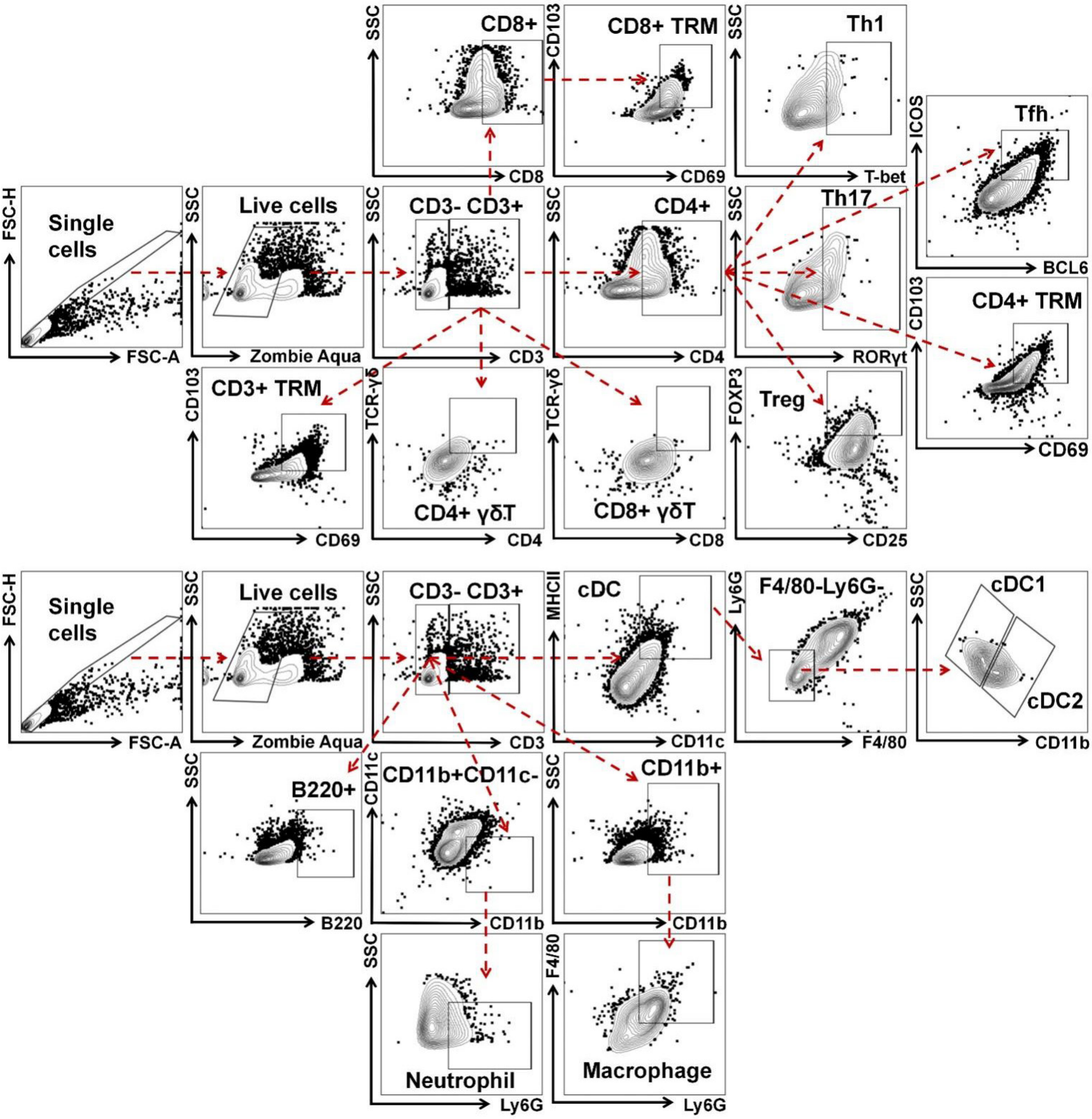

Figure S5. Flow cytometry gating strategy of skin lesions.

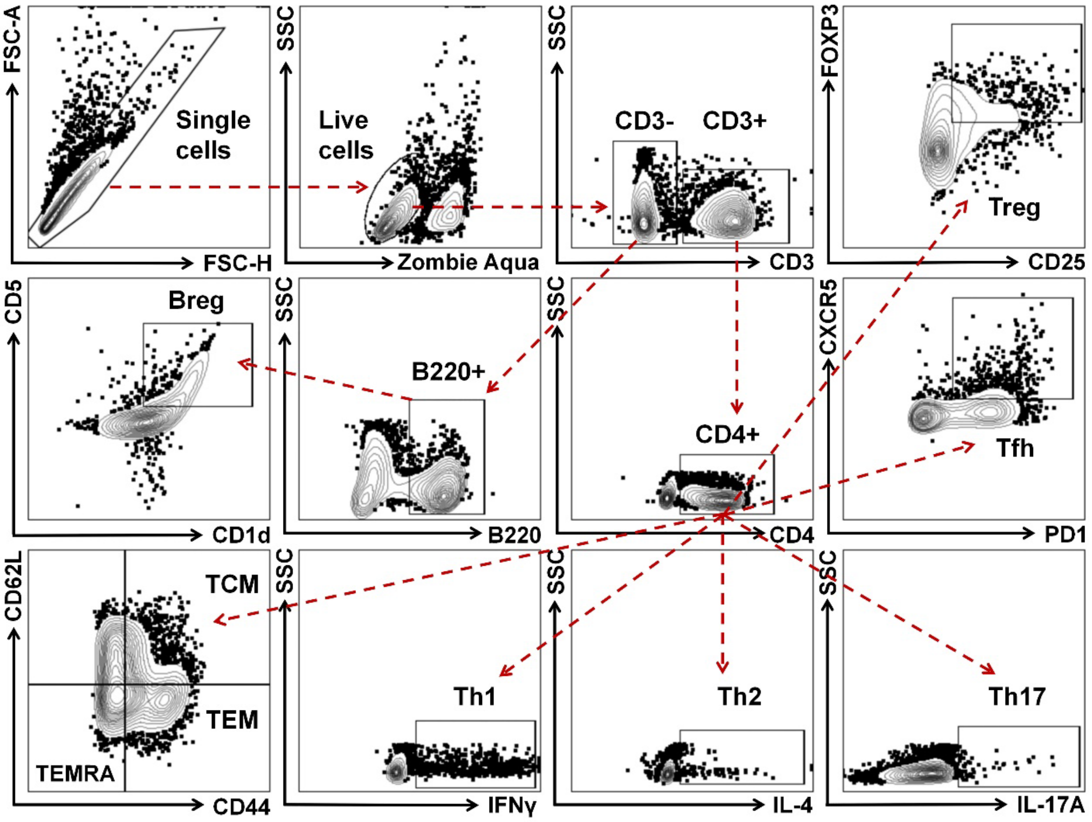

Figure S6. Flow cytometry gating strategy of spleens.
